# Alternate Strategies to Induce Dynamically Modulated Transient Transcription Machineries

**DOI:** 10.1021/acsnano.3c05336

**Published:** 2023-09-05

**Authors:** Zhenzhen Li, Jianbang Wang, Itamar Willner

**Affiliations:** The Institute of Chemistry, The Center for Nanoscience and Nanotechnology, The Hebrew University of Jerusalem, Jerusalem 91904, Israel

**Keywords:** DNAzyme, strand displacement, nickase, gated transcription, dissipative circuit, RNA aptamer

## Abstract

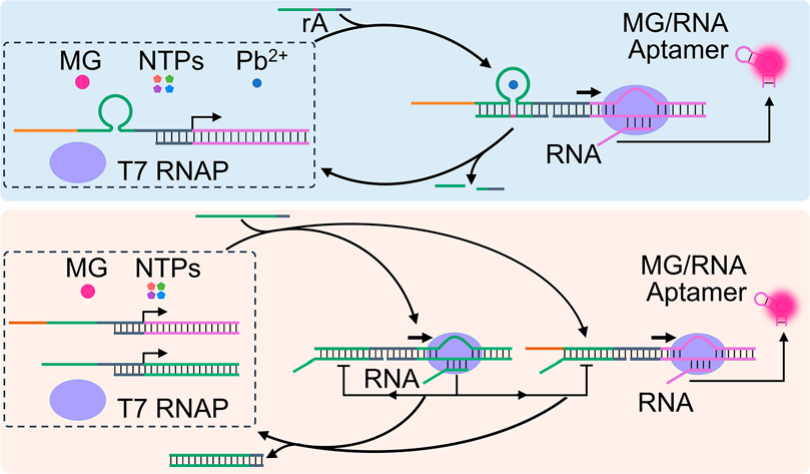

Emulating native transient transcription machineries modulating temporal gene expression by synthetic circuits is a major challenge in the area of systems chemistry. Three different methods to operate transient transcription machineries and to modulate the gated transcription processes of target RNAs are introduced. One method involves the design of a reaction module consisting of transcription templates being triggered by promoter fuel strands transcribing target RNAs and in parallel generating functional DNAzymes in the transcription templates, modulating the dissipative depletion of the active templates and the transient operation of transcription circuits. The second approach involves the application of a reaction module consisting of two transcription templates being activated by a common fuel promoter strand. While one transcription template triggers the transcription of the target RNA, the second transcription template transcribes the anti-fuel strand, displacing the promoter strand associated with the transcription templates, leading to the depletion of the transcription templates and to the dynamic transient modulation of the transcription process. The third strategy involves the assembly of a reaction module consisting of a reaction template triggered by a fuel promoter strand transcribing the target RNA. The concomitant nickase-stimulated depletion of the promoter strand guides the transient modulation of the transcription process. Via integration of two parallel fuel-triggered transcription templates in the three transcription reaction modules and application of template-specific blocker units, the parallel and gated transiently modulated transcription of two different RNA aptamers is demonstrated. The nickase-stimulated transiently modulated transcription reaction module is applied as a functional circuit guiding the dynamic expression of gated, transiently operating, catalytic DNAzymes.

Transient interactions between transcription factors or hormones and the transcription machinery play key roles in the dynamic modulation of gene expression^1,2^ and secondary physiological processes such as differentiation and development,^3,4^ and spatially and temporally misregulated transcription programs may lead to diverse diseases.^5,6^ The field of “systems chemistry” aims to emulate the complexity of natural processes by chemical principles, with the vision that such biomimetic systems could be harnessed to provide innovative diagnostic and therapeutic means, alternative energy sources, and creative functional materials.^7,8^ Within these efforts, the development of dynamic supramolecular networks revealing adaptive,^9−11^ hierarchically adaptive,^12^ and feedback mechanisms^13^ and the design of transient, out-of-equilibrium systems^14−16^ attracted growing interests, and the basic concepts of dynamic supramolecular chemistry were applied to develop functional materials (gels),^17^ structures,^18,19^ and catalysts.^20^ Particularly, the information encoded in nucleic acids, reflected by dynamic signal-triggered reconfiguration of the biopolymer,^21^ sequence-guided recognition properties (aptamers),^22,23^ catalytic functions (DNAzymes),^24,25^ and the sequence-specific reactivity features of nucleic acids, in the presence of enzymes (endonucleases, nickases),^26−29^ were broadly used to develop nucleic acid-based constitutional dynamic networks (CDNs)^30^ and dissipative, transient nucleic acid reaction modules.^31−33^ Nucleic acid-based CDNs revealing adaptive,^34,35^ hierarchically adaptive,^36^ feedback and intercommunication features^37,38^ were reported. These CDNs systems were applied to assemble hydrogel materials revealing dynamic stiffness properties for self-healing and controlled drug release,^39^ operation of dynamic biocatalytic cascades^40^ and controlled catalysis, drug release, and functions of nanoparticles.^41,42^ In addition, dissipative, transient nucleic acid reaction frameworks were applied for the dynamic, transient release of loads,^43^ operation of transient biocatalytic cascades,^44^ formation of nanostructures, and control over optical properties of nanoparticles.^45,46^

Emulating the dynamic features of native transcription machineries by synthetic nucleic acids is still in its infancy, yet progress to advance facets related to dynamic transcription and gene expression has been demonstrated. This includes the design of transcriptional oscillators,^47−49^ transcriptional switches,^50^ bistable regulatory networks,^51^ programmed switchable transcription within topologically constrained transcription templates,^52^ and constitutional dynamic networks guiding dynamic gene expression and protein translation.^53^ Moreover, with the advances to construct artificial cells, i.e., “protocells”,^54−56^ the need to integrate transcription machineries into cell-like containments and to dictate complex spatiotemporal dynamic transcription processes within the artificial cell is a challenging goal. Indeed, recent studies reported on the integration and activation of a transcription machinery in a fused liposome assembly^57^ and the triggered topologically switchable operation of a transcription template within a hydrogel microcapsule, cell-like containment.^58^ All of these processes are, however, far beyond the desired spatiotemporal transcription complexity where transient gated, fan-out, and cascaded transcription processes are desirable.

In fact, in order to enhance the complexity of biomimetic transcription machineries (e.g., gated or cascaded processes), it is essential to develop dynamically controlled, transiently operating transcription templates. Three general methods to engineer temporally operating transcription templates were reported. By one method^51,59^ the transcription template was activated by a DNA-fuel, and the active DNA template was deactivated by displacement with an RNA anti-fuel that was degraded by RNase to reactivate the fuel strand. The second approach^60^ employed a reaction module consisting of a transcription template that was triggered by the ribonucleoside triphosphate (NTP) fuels to yield a RNA product that resulted in the structural displacement of the reaction module that led to the formation of a catalytically active DNAzyme and an intermediate RNA/DNA duplex that was digested by RNase, leading to the transient dissipative formation and depletion of the DNAzyme intermediate product. The third method^61^ involved the design of a reaction module consisting of two transcription templates and an inhibited aptamer/T7 RNA polymerase (RNAP) complex. The fuel-driven separation of the aptamer/RNAP complex activated the RNAP/NTPs operation of the two transcription templates, where one led to the target RNA and the second template transcribed the inhibiting T7 RNAP aptamer, leading to the temporal transient inhibition of the central transcription machinery. These methods to stimulate the transient operation of transcription machineries suffer, however, from basic limitations, preventing the possibility to develop complex transcription pathways. While the RNase-based systems consume the RNA products, thus limiting their subsequent uses, the transcription-induced generation of an inhibiting RNAP aptamer introduces a competitive pathway that utilizes most of the NTPs fuels, thus prohibiting the economic use of the fuels for complex transcription processes.

Here we introduce three alternative strategies to promote transient transcription machineries overcoming the outlined limitations associated with the RNase systems or the RNAP inhibiting concept. These include the application of a DNAzyme-functionalized transcription template, a strand displacement dynamic inhibition of the transcription template, and a nickase-controlled transient operation of the transcription template. Besides the introduction of alternative mechanisms to operate transient transcription machineries, the systems are employed to guide gated transcription processes. Moreover, we present the possible use of transiently operating transcription machineries to assemble gated, transiently operating catalytic systems (DNAzymes).

## Results and Discussion

**Figure 1:**
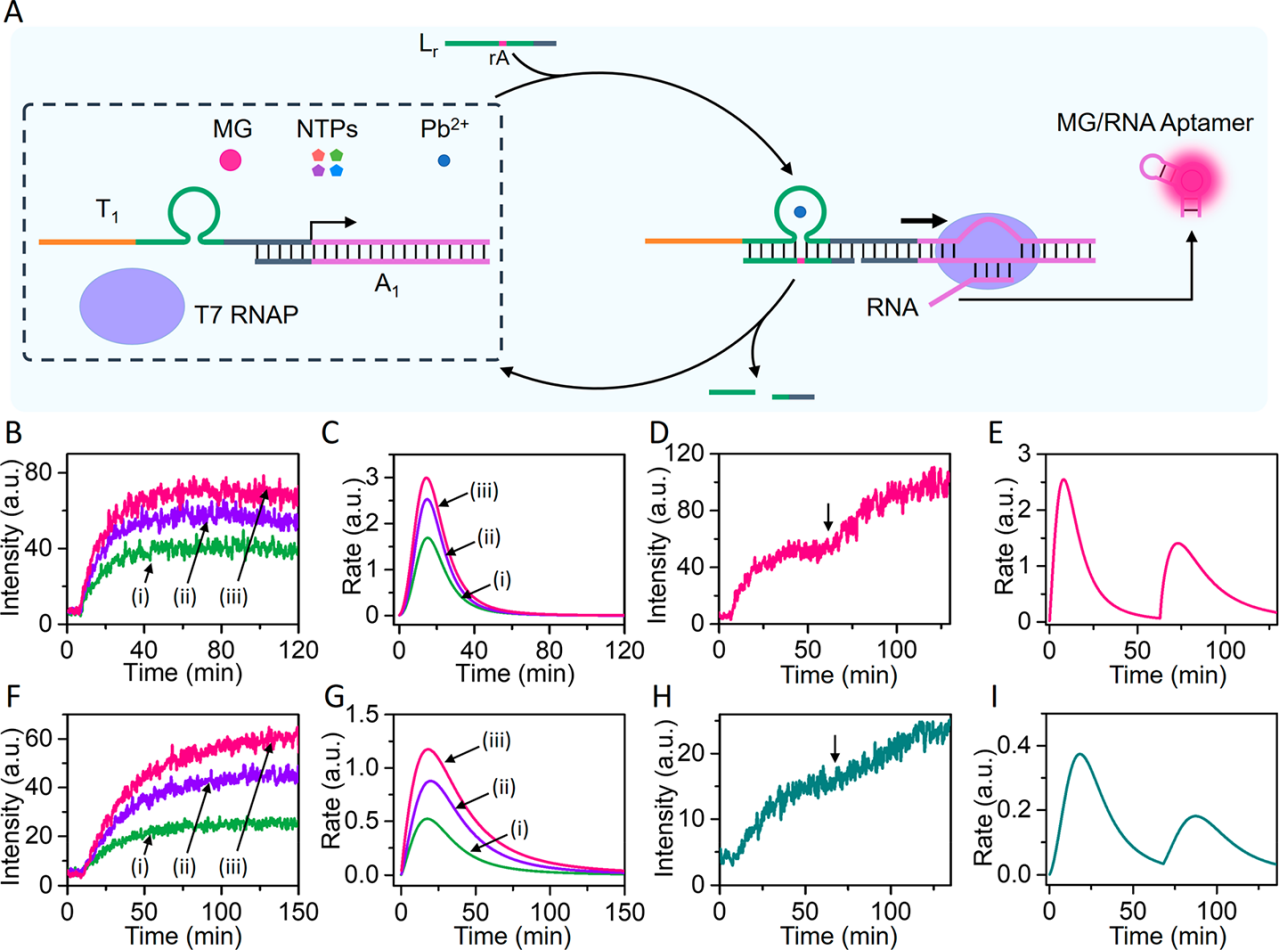
(A) Schematic reaction module for the DNAzyme-triggered operation of a transient transcription machinery synthesizing the MG-RNA aptamer. (B) Temporal fluorescence intensities of the MG-RNA aptamer generated by the transient reaction module shown in (A) in the presence of variable concentrations of the fuel triggering strand L_r_ (T_1_/A_1_, 0.1 μM; T7 RNAP, 1.25 × 10^3^ U/mL; MG, 4 μM; NTPs, 4 mM each; Pb^2+^ ions, 0.1 μM): (i) 0.05 μM, (ii) 0.1 μM, and (iii) 0.15 μM. (C) Time-dependent catalytic transcription rates corresponding to the transient synthesis of the MG-RNA aptamer in the presence of different concentrations of L_r_: (i) 0.05 μM, (ii) 0.1 μM, and (iii) 0.15 μM. (D) Temporal fluorescence intensities upon the cyclic operation of the transient reaction module transcribing the MG-RNA aptamer. The time marked with an arrow indicates the time reactivation of the reaction module by adding the fuel strand L_r_: 0.1 μM and 0.5 μM (T_1_/A_1_, 0.1 μM; T7 RNAP, 1.25 × 10^3^ U/mL; MG, 4 μM; NTPs, 4 mM each; Pb^2+^ ions, 0.1 μM). (E) Cyclic catalytic rates corresponding to the stepwise operation of the transient transcription machinery. (F) Temporal fluorescence changes corresponding to the DNAzyme-triggered operation of the transcription machinery shown in Figure S8 synthesizing the DFHBI-RNA aptamer in the presence of variable concentrations of the fuel triggering strand L_r_ (T_2_/A_2_, 0.2 μM; T7 RNAP, 1.875 × 10^3^ U/mL; DFHBI, 8 μM; NTPs, 4 mM each; Pb^2+^ ions, 0.1 μM): (i) 0.1 μM; (ii) 0.2 μM; (iii) 0.3 μM. (G) Transient catalytic transcription rates corresponding to the DFHBI-RNA aptamer (derivatives of the curves shown in (F)). (H) Temporal fluorescence changes upon cyclic operation of the transient reaction module transcribing the DFHBI-RNA aptamer. The time marked with an arrow indicates the time reactivation of the reaction module by adding the fuel strand L_r_: 0.1 μM and 0.2 μM (T_2_/A_2_, 0.1 μM; T7 RNAP, 1.875 × 10^3^ U/mL; DFHBI, 8 μM; NTPs, 4 mM each; Pb^2+^ ions, 0.1 μM). (I) Cyclic transcription catalytic rates corresponding to the stepwise operation of synthesizing the DFHBI-RNA aptamer.

The first system to assemble a transient dissipative transcription machinery is displayed in Figure 1(A) and involves a DNAzyme as a catalytic regulator controlling the dissipative transient operation of the transcription template. Specifically, the use of the Pb^2+^-ion-dependent DNAzyme and the transient transcription of the Malachite Green (MG) aptamer or the ((5*Z*)-5-[(3,5-difluoro-4-hydroxyphenyl)methylene]-3,5-dihydro-2,3-dimethyl-4*H*-imidazol-4-one) (DFHBI) broccoli aptamer are presented. The reaction module consists of the inactive T_1_/A_1_ template, where T_1_ includes the sequence-specific Pb^2+^-ion binding loop and the respective substrate-binding arms and the strand A_1_ is engineered to yield upon transcription the MG-RNA aptamer. T7 RNAP, MG, the NTPs mixture, and Pb^2+^ ions are included in the reaction module. Under these conditions, the transcription template is incomplete and the transcription process is blocked. Subjecting the reaction module to the ribonucleobase-modified strand L_r_ acting as a promoter complementing the transcription template, yet engineered to act as substrate of the DNAzyme, results in the activation of the transcription machinery and the expression of the MG-RNA aptamer. The binding of the promoter to the transcription machinery and the triggered formation of the MG aptamer, however, lead to the concomitant Pb^2+^-ion-dependent DNAzyme-induced cleavage of L_r_, resulting in the degradation of the promoter unit to waste products and their separation from the transcription template, leading to the recovery of the rest inactive reaction module and to the temporal, transient transcription of the MG aptamer. The temporal synthesis of the MG-RNA aptamer is, then, followed by the temporal fluorescence changes of the MG aptamer complex. Figure 1(B) depicts the temporal fluorescence changes corresponding to the MG aptamer complex (λ_ex_ = 635 nm and λ_em_ = 665 nm) upon subjecting the reaction module to different concentrations of L_r_. As the concentration of L_r_ increases, the time-dependent fluorescence changes are intensified, consistent with increased concentration of the active transcription template synthesizing the MG-RNA aptamer. The time-dependent fluorescence changes decrease with time and reach saturation values consistent with the DNAzyme-stimulated blockage of the transcription machinery and transient recovery of the inactive reaction module. (For the formulation of a kinetic model and the simulation of the temporal fluorescence changes of the DNAzyme-triggered transient transcription of the MG-RNA aptamer, in the presence of different fuel concentrations, see Figures S1–S3 and Table S1 in the Supporting Information and accompanying discussions.) The dynamic temporal fluorescence changes of the MG aptamer complex generated by the reaction module were then translated into catalytic rates forming the MG-RNA aptamer (derivatives of the time-dependent fluorescence changes shown in Figure 1(B)), and these are displayed in Figure 1(C) where transient, dissipative transcription rates are observed. The transcription rates reach a maximum value after ca. 18 min; afterward, time-dependent DNAzyme-guided depletion of the transcription processes is observed, and after ca. 40 min the inactive reaction modules are recovered and the formation of the MG-RNA aptamer is blocked. (For duplicate experimental results of Pb^2+^-DNAzyme-modulated transcription machinery, see Figure S4. For a control experiment of Pb^2+^-DNAzyme-modulated transcription machinery demonstrating the inhibition of lack of leakage of the transcription machinery, in the absence of the fuel strand L_r_, see Figure S5, Supporting Information. For the temporal fluorescence intensities of the MG-RNA aptamer complex generated by the transcription machinery in the presence of different concentrations of NTPs, see Figure S6.) By re-addition of the triggering ribonucleobase-modified promoter L_r_, the transient, temporal reactivation of the transcription machinery was demonstrated, Figure 1(D) and (E). (For additional control experiments characterizing the Pb^2+^-ion-dependent DNAzyme-controlled transcription of the MG aptamer, see Figure S7 and the accompanying discussion.)

The same concept was applied to design an alternate Pb^2+^-ion-dependent DNAyzme-guided transient transcription machinery synthesizing the DFHBI-RNA aptamer. In this case, a reaction module consisting of transcription template T_2_/A_2_, T7 RNAP, DFHBI, and NTPs was assembled, Figure S8. The strand A_2_ included an engineered sequence for transcription of the DFHBI-RNA aptamer. The reaction module was triggered with the ribonucleobase-modified promoter L_r_, and the temporal transient transcription of the DFHBI-RNA aptamer was followed by time-dependent fluorescence changes of the DFHBI aptamer complex (λ_ex_ = 470 nm and λ_em_ = 500 nm). Figure 1(F) shows the time-dependent fluorescence intensities of the DFHBI aptamer complex upon triggering the reaction module with different concentrations of L_r_, and Figure 1(G) depicts the transient catalytic rates for generating the DFHBI aptamer in the presence of different concentrations of L_r_. Slightly slower DNAzyme-driven depletion rates of this transcription machinery, as compared to the MG aptamer transcription machinery are observed (the recovery of the inactive reaction module and blockage of the transcription machineries are observed after ca. 75 min). As before, by re-addition of the triggering strand L_r_, the transient, DNAzyme-guided transcription machinery was reactivated, Figure 1(H) and (I).

**Figure 2:**
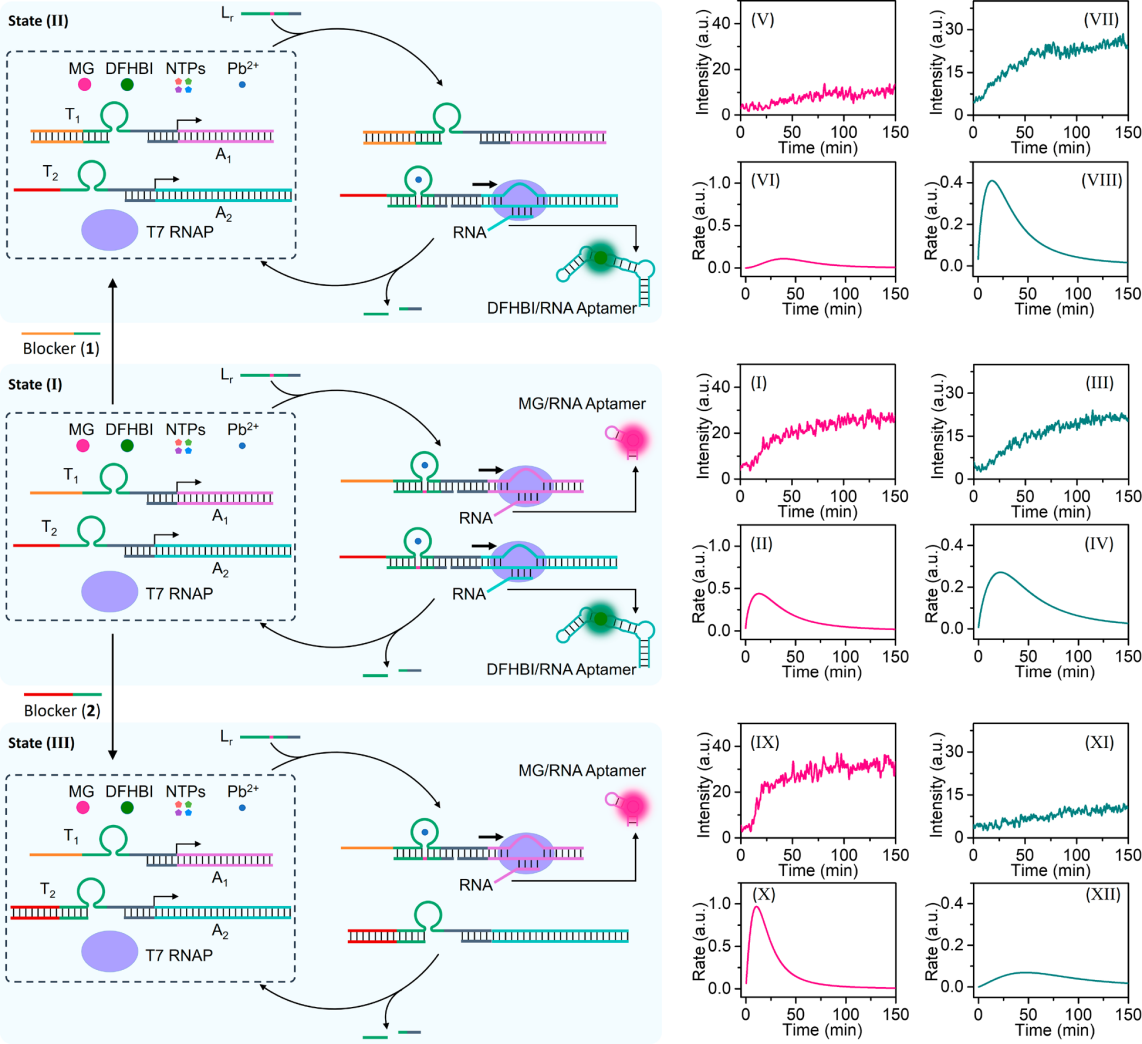
Schematic assembly of a reaction module, State I, leading to the gated DNAzyme-triggered transient transcription of two different RNA aptamer products, e.g., MG-RNA aptamer and/or DFHBI aptamer, in the presence of two blocker strands. The reaction module consists of two transcription templates, T_1_/A_1_ (0.05 μM) and T_2_/A_2_ (0.2 μM), T7 RNAP (1.25 × 10^3^ U/mL), NTPs (4 mM each), Pb^2+^ ions (0.1 μM), MG (4 μM), and DFHBI (8 μM). In the presence of L_r_ (0.1 μM) as fuel strand, the DNAzyme-controlled two transcription machineries are activated, leading to the temporal, transient transcription of the MG-RNA aptamer and DFHBI-RNA aptamer. The temporal fluorescence intensities and the respective catalytic rates for the formation of the MG-RNA aptamer and DFHBI-RNA aptamer are displayed in Panels I/II and III/IV, respectively. Subjecting the reaction module in State I to blocker **1** (2 μM), leading to State II, where template T_1_/A_1_ is inhibited, leading to the gated operation of the T_2_/A_2_ transcription machinery. The gated transcription of the MG-RNA aptamer is inhibited, Panels V/VI, while the DFHBI-RNA aptamer proceeds, reflected by the selective temporal fluorescence changes of the DFHBI aptamer complex and the accompanying time-dependent catalytic rates corresponding to the formation of DFHBI aptamer, Panels VII/VIII. Interaction of the reaction module in State I with blocker **2** (4 μM), yield State III, where the template T_2_/A_2_ is selectively inhibited, leading to the gated DNAzyme-triggered transient transcription of the MG-RNA aptamer. This is reflected by gated temporal fluorescence changes of the MG aptamer complex and the respective time-dependent catalytic rates corresponding to the formation of the MG aptamer, Panels IX/X, while the transcription of the DFHBI-RNA aptamer is inhibited, Panels XI/XII.

The DNAzyme-guided transient operation of the transcription machineries was then applied to assemble a gated transcription reaction module, Figure 2. The reaction module consisted of a mixture of transcription templates T_1_/A_1_ and T_2_/A_2_ that included the Pb^2+^-ion-dependent DNAzyme loops as functional units. T7 RNAP, NTPs, MG, DFHBI, and Pb^2+^ ions were included in the reaction module. In the presence of the common triggering promoter strand L_r_, the Pb^2+^-ion-dependent DNAzyme-controlled transient transcription templates T_1_/A_1_ and T_2_/A_2_ are activated, leading to the concomitant formation of the MG and DFHBI aptamers reflected in the temporal fluorescence intensities of the MG aptamer and DFHBI aptamer complexes and the accompanying transient catalytic rates generating the aptamers, Figure 2, Panels I–IV. Treatment of the reaction module with blocker **1**, results in the hybridization of the blocker **1** with the DNAzyme tether unit associated with T_1_. The tight duplex between blocker **1** and T_1_ prohibits binding of the promoter unit, L_r_, to T_1_. Subjecting the reaction module, consisting of the **1**-blocked configuration, State II, to the ribonucleobase-modified strand L_r_ activates the gated transcription of template T_2_/A_2_ while the operation of template T_1_/A_1_ is switched off. This is reflected by the Pb^2+^-ion-dependent DNAzyme guided transient fluorescence changes of the DFHBI aptamer complex, Figure 2, Panel VII, and the accompanying transient catalytic rates of the synthesis of the DFHBI-RNA aptamer, Figure 2, Panel VIII, while the formation of the MG-RNA aptamer is blocked. Subjecting the reaction module in State I to the blocker strand **2**, results in, however, the selective blocking of template T_2_/A_2_, State III, leading to the gated transcription of the MG-RNA aptamer. This is reflected with the selective, switched-on, temporal fluorescence changes of the MG aptamer complex, Figure 2, Panel IX, and the concomitant transient catalytic rates, generating the MG-RNA aptamer, Figure 2, Panel X, while under these conditions, the formation of the DFHBI-RNA aptamer is almost switched off, Figure 2, Panels XI and XII.

**Figure 3:**
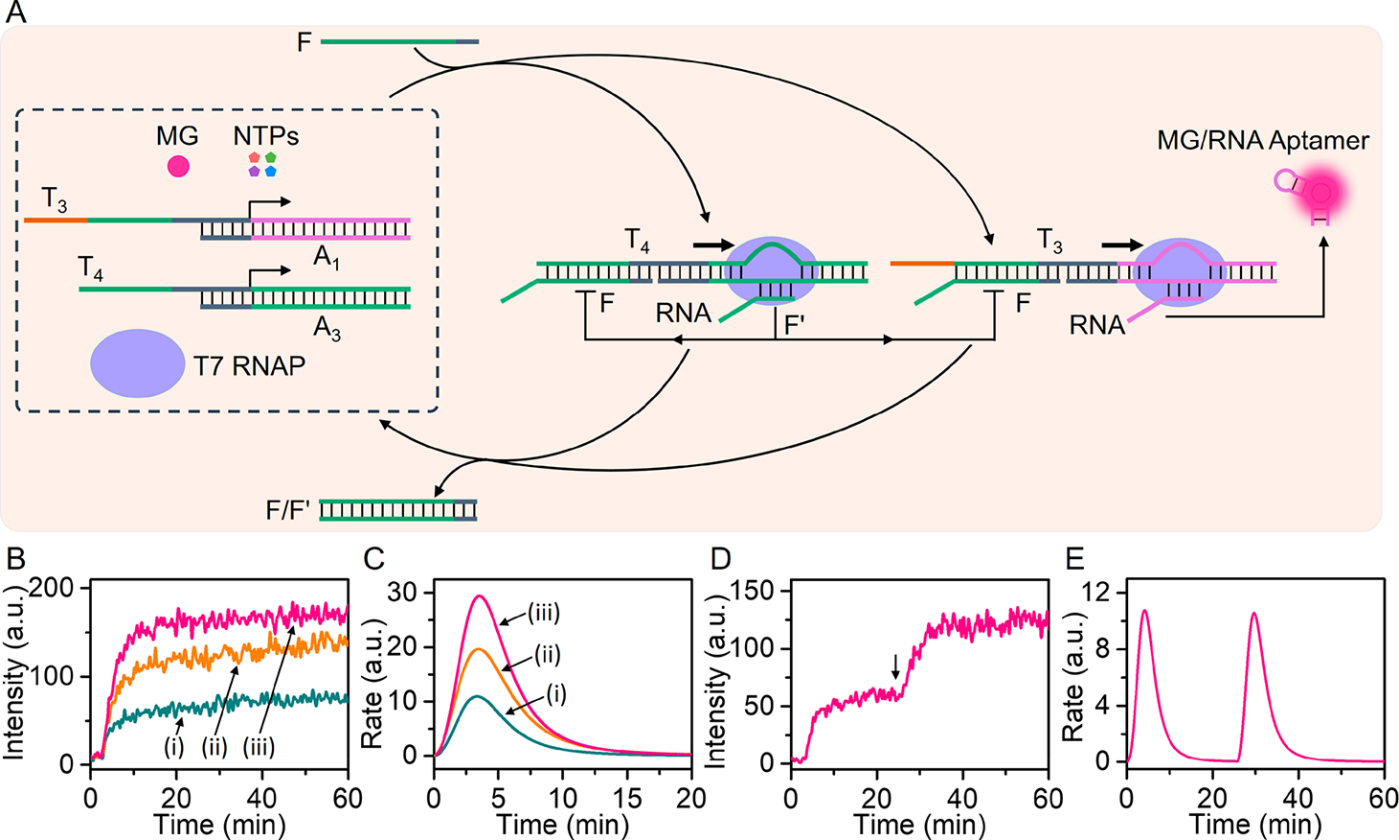
(A) Schematic configuration of a reaction module leading to the transient operation of a transcription machinery synthesizing the MG-RNA aptamer through triggered activation of the transcription template with a fuel strand and the competitive displacement of the fuel strand by an anti-fuel strand transcribed by an auxiliary transcription machinery present in the reaction module. (B) Temporal fluorescence changes corresponding to the transcribed MG-RNA aptamer following scheme shown in (A) in the presence of variable concentrations of fuel F (T_3_/A_1_, 0.05 μM; T_4_/A_3_, 0.05 μM; T7 RNAP, 2.5 × 10^3^ U/mL; MG, 4 μM; NTPs, 4 mM each): (i) 0.05 μM; (ii) 0.1 μM; (iii) 0.15 μM. (C) Time-dependent catalytic transcription rates corresponding to the transient synthesis of the MG-RNA aptamer in the presence of different concentrations of fuel F: (i) 0.05 μM; (ii) 0.1 μM; (iii) 0.15 μM. (D) Temporal fluorescence changes upon cyclic operation of the transient reaction module. The time marked with an arrow indicates the time reactivation of the reaction module by adding the fuel strand F: 0.033 μM and 0.33 μM (T_3_/A_1_, 0.05 μM; T_4_/A_3_, 0.05 μM; T7 RNAP, 2.5 × 10^3^ U/mL; MG, 4 μM; NTPs, 4 mM each). (E) Cyclic catalytic rates corresponding to the stepwise operation of the transient transcription machinery.

**Figure 4:**
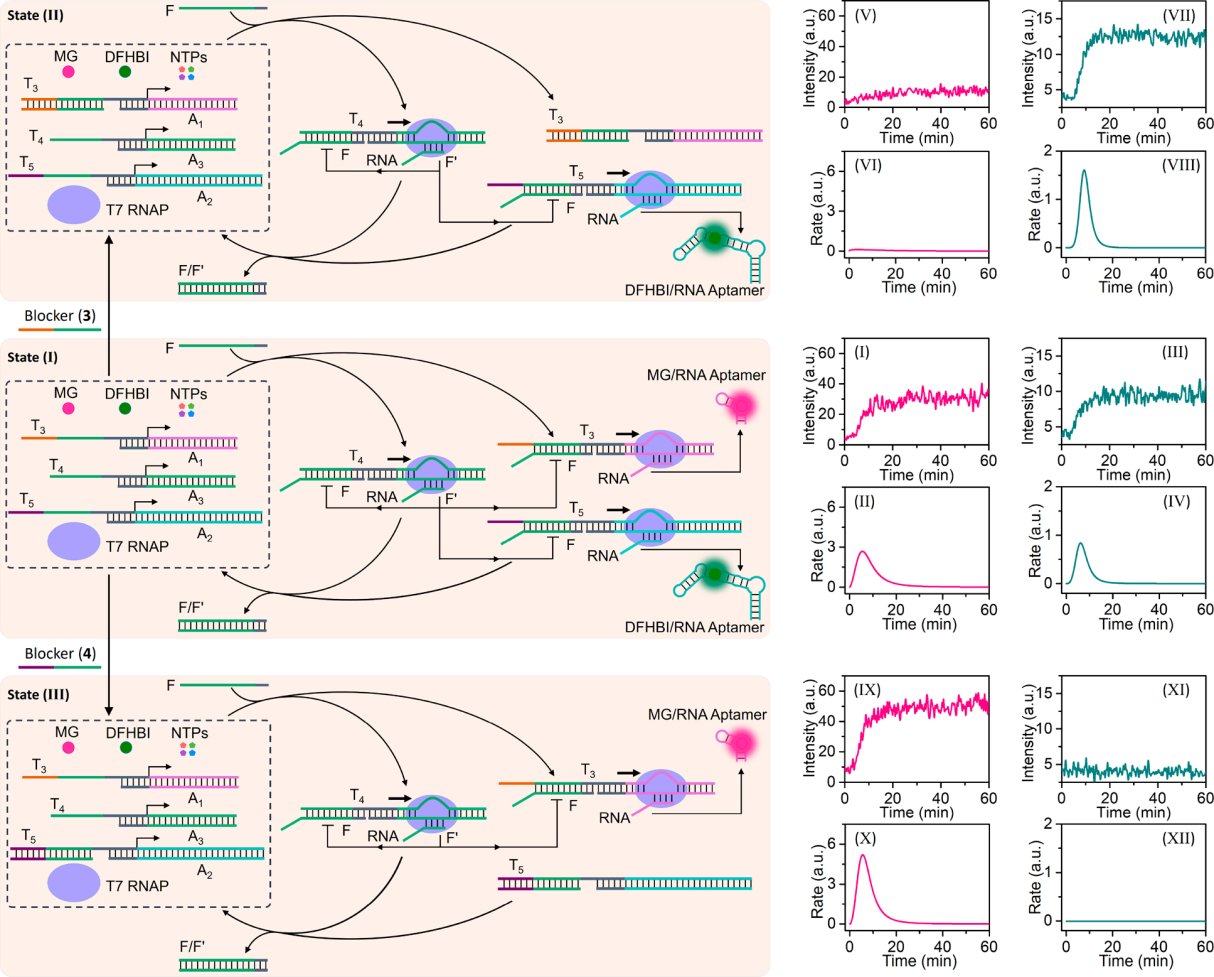
Schematic configuration of a reaction module in State I, leading to the gated transient operation of two transcription machineries synthesizing the MG-RNA aptamer and the DFHBI-RNA aptamer, using the strand displacement principle to guide the dissipative temporal activities of the transcription machineries. The temporal fluorescence changes of the MG-RNA aptamer and the DFHBI-RNA aptamer and the accompanying transient catalytic rates associated with the operation of the transcription template T_3_/A_1_ and T_5_/A_2_ are displayed in Panels I/II and III/IV, respectively (T_3_/A_1_, 0.02 μM; T_4_/A_3_, 0.03 μM; T_5_/A_2_, 0.06 μM; T7 RNAP, 2.06 × 10^3^ U/mL; MG, 4 μM; DFHBI, 8 μM; NTPs, 4 mM each; F, 0.03 μM). Subjecting the reaction module in State I to blocker **3** (0.2 μM) yields the reaction module in State II where the template T_3_/A_1_ is blocked, Panels V/VI, leading to the gated transcription of the DFHBI-RNA aptamer. The gated, selective, temporal fluorescence changes of the transcribed DFHBI aptamer complex and the transient catalytic rates of the transcription of the DFHBI-RNA aptamer are displayed in Panels VII/VIII. Treatment of the reaction module in State I with blocker **4** (0.3 μM) yields State III, where the triggered gated transcription of the MG-RNA aptamer proceeds. The temporal fluorescence changes of the MG aptamer complex and the transient catalytic rates associated with transcription of the MG aptamer are displayed in Panels IX/X, while the transcription of the DFHBI aptamer is inhibited, Panels XI/XII.

The second approach to trigger the transcription apparatus and to stimulate the gated operation of the transcription machineries is displayed in Figure 3 and Figure 4, and it involves the strand-displacement of the promoter strand as a functional motif to regulate the dynamic, transient operation of the transcription process. The reaction module, Figure 3(A), consists of two incomplete, inactive transcription templates T_3_/A_1_ and T_4_/A_3_, T7 RNAP, NTPs, and the MG ligand. Subjecting the reaction module to the fuel strand F activates the two transcription machineries. While activation of T_3_/A_1_ leads to the transcription of the MG-RNA aptamer as product, activation of the template T_4_/A_3_ leads to the transcription of the RNA product F′ that acts as an anti-fuel strand displacing the activator strand F. That is, formation of F/F′ duplex regenerates the rest reaction module, leading to the transient operation of the MG aptamer transcription machinery. Figure 3(B) depicts the temporal fluorescence changes generated by MG aptamer complex in the presence of different concentrations of the fuel strand F. The fluorescence intensities reach saturation levels, consistent with the F′-temporal blockage of the transcription machineries. As the concentration of F increases, the rate of MG aptamer formation increases and the saturation level of the resulting MG aptamer is higher. (For the formation of a kinetic model and the simulation of the temporal fluorescence changes of the strand-displacement-stimulated transient transcription of the MG-RNA aptamer, in the presence of different fuel concentrations, see Figures S9 and S10 and Table S2 in the Supporting Information and accompanying discussion.) Figure 3(C) depicts the temporal rates corresponding to the formation of the MG aptamer, at variable concentrations of F, where dissipative, transient transcription rates are observed. (For duplicate experimental results of strand-displacement-stimulated transcription machinery, see Figure S11. For a control experiment of strand-displacement-stimulated transcription machinery suggesting the inhibition of leakage of the transcription machinery in the absence of the fuel strand F, see Figure S12. For additional experiments probing the effect of different concentrations of the NTPs and variable concentrations of the template T_4_/A_3_, see Figures S13 and S14. For further electrophoretic experiments supporting the transient temporal operation of the dual transcription machineries, see Figure S15 and accompanying discussion. For additional control experiments employing a triggering strand, Fc, not being displaced by the transcribed F′, and an experiment probing the stability of template T_4_/A_3_ in the presence of fuel strand F, see the Supporting Information, Figures S16 and S17 and accompanying discussions.) Figure 3(D) and (E) depicts the transient, cyclic reactivation of the reaction module by re-addition of the fuel strand F (time marked with arrow) after the synthesis of MG aptamer reached saturation and the reaction module is reactivated. (For the assembly and operation of an analog strand-displacement-guided reaction module leading to a transient transcription machinery synthesizing the DFHBI-RNA aptamer, see Figures S18–S20.)

Figure 4 presents the application of the fuel-strand-guided activation of two transcription machineries and the application of the transcription-guided strand displacement principle for temporal, transient activation of the two transcription machineries yielding gated transient synthesis of the MG aptamer or the DFHBI aptamer complexes. The reaction module consists of three incomplete, inactive, reaction templates T_3_/A_1_, T_4_/A_3_, and T_5_/A_2_, the T7 RNAP, NTPs, and the ligands MG and DFHBI, State I. Subjecting the reaction module in State I to the fuel strand F, activates all three templates. The activation of the two templates T_3_/A_1_ and T_5_/A_2_ leads to the transcription of the MG-RNA aptamer and the DFHBI aptamer, followed by time-dependent fluorescence changes of the MG aptamer complex and the DFHBI aptamer complex. The concomitant activation of the template T_4_/A_3_ leads to the transcription of the strand F′ that displaces the activating strand F associated with T_3_, T_4_, and T_5_, resulting in the depletion of the active templates and to the transient synthesis of the MG aptamer and DFHBI aptamer products. Figure 4, Panels I and III, shows the temporal fluorescence changes associated with transient formation of the MG aptamer and DFHBI aptamer complexes, and the temporal transient rates corresponding to the formation of the MG aptamer and DFHBI aptamer complexes are displayed in Panels II and IV, by the reaction module in State I. Interaction of the reaction module in State I with the blocker unit **3** leads to the hybridization of the blocker with the template T_3_/A_1_. Under these conditions, in the presence of the fuel strand F, only the transcription templates T_5_/A_2_ and T_4_/A_3_ are activated, resulting in the transient transcription of the DFHBI aptamer and the blockage of the MG aptamer transcription template, T_3_/A_1_, State II. Panels V and VII show the temporal fluorescence changes associated with the formation of the MG aptamer and the DFHBI aptamer by the reaction module in State II. While the formation of the MG aptamer is blocked, effective temporal fluorescence changes of the DFHBI aptamer complex that levels off after ca. 15 min are observed. Similarly, treatment of the reaction module in State I with the blocker unit **4** yields the reaction module in State III, where template T_5_/A_2_ is blocked, and the gated triggered activation of the template T_3_/A_1_ and T_4_/A_3_ by the fuel strand F proceeds. Panels IX and XI depict the experimental results demonstrating the temporal increase and leveling off of the fluorescence intensities of the MG aptamer complex, while the fluorescence of the DFHBI aptamer complex is blocked. Similarly, Panels X and XII show the gated transient time-dependent rates of formation of the MG or DFHBI aptamer by State III. Evidently, the integration of two transcription machineries activated by a common fuel activating strand and an auxiliary transcription machinery activated by the fuel strand to yield an anti-fuel strand displacing the activating strands, associated with all transcription templates, leads to the competitive dissipation of all transcription machineries. In the presence of appropriate blocker units, gated operation of the transcription templates is demonstrated.

**Figure 5:**
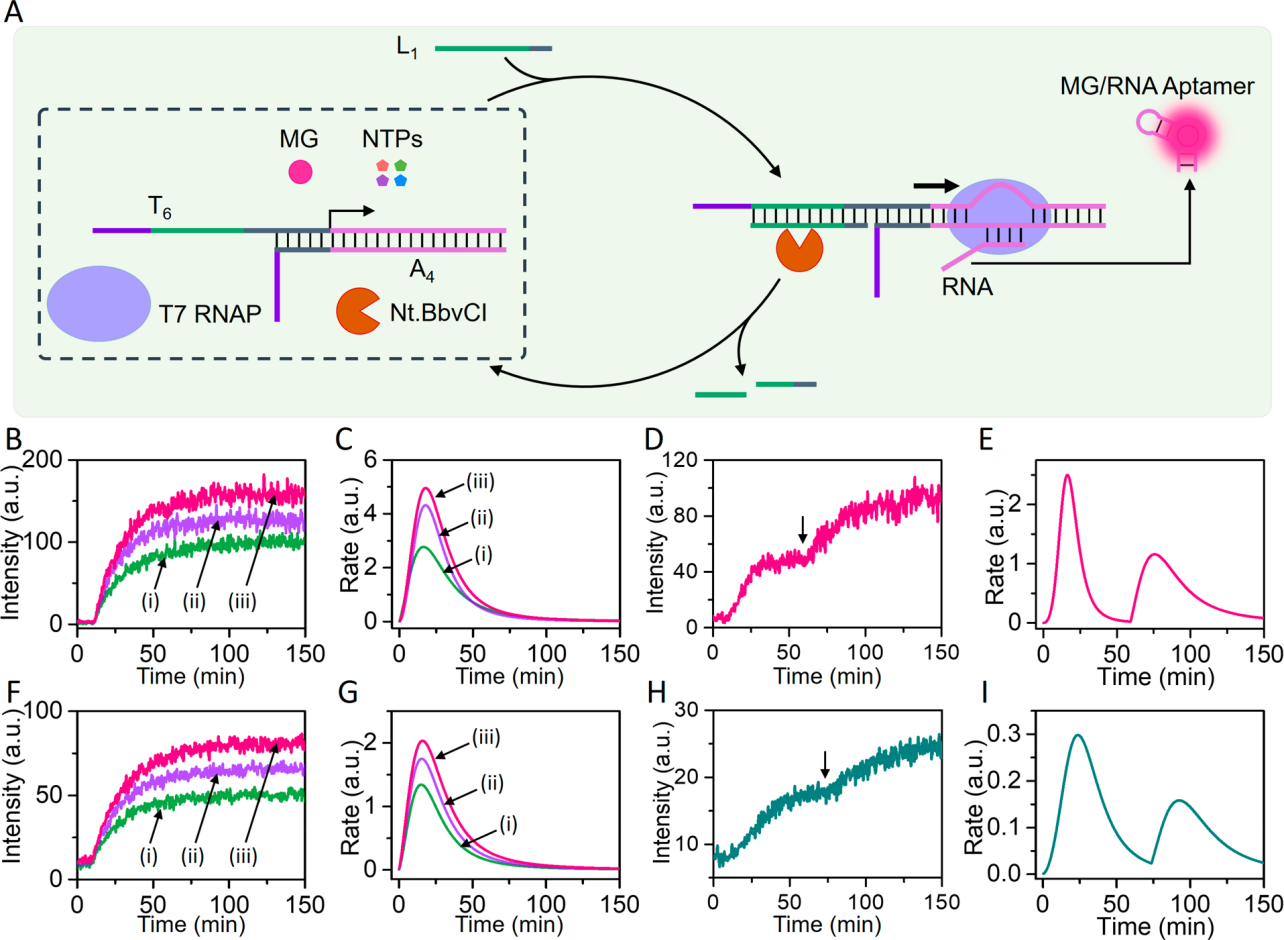
(A) Schematic reaction module corresponding to the nickase-stimulated transient operation of a transcription machinery forming the MG-RNA aptamer. (B) Temporal fluorescence changes corresponding to the transcription of the MG-RNA aptamer upon the triggered nickase-stimulated transcription machinery, in the presence of variable concentrations of the trigger L_1_ (T_6_/A_4_, 0.02 μM; T7 RNAP, 1.25 × 10^3^ U/mL; Nt.BbvCI, 83.3 U/mL; MG, 4 μM; NTPs, 4 mM each): (i) 0.1 μM, (ii) 0.2 μM, and (iii) 0.3 μM. (C) Transient time-dependent catalytic rates corresponding to the transcription of the MG-aptamer in the presence of different concentrations of L_1_: (i) 0.1 μM, (ii) 0.2 μM, and (iii) 0.3 μM. (D) Temporal fluorescence changes upon cyclic stepwise operation of the nickase-stimulated operation of the transcription of the MG-aptamer by adding the fuel strand L_1_: 0.02 μM and 0.1 μM (T_6_/A_4_, 0.02 μM; T7 RNAP, 1.25 × 10^3^ U/mL; Nt.BbvCI, 83.3 U/mL; MG, 4 μM; NTPs, 4 mM each). (Arrow indicates time-interval use; the trigger L_1_ activates the second transient transcription cycle). (E) Time-dependent catalytic rates upon the stepwise operation of the transcription cycles of the MG-RNA aptamer. (F) Temporal fluorescence changes upon transcription of the DFHBI-RNA aptamer following the reaction module shown in Figure S27 using different concentrations of the trigger L_2_ (T_7_/A_5_, 0.04 μM; T7 RNAP, 1.25 × 10^3^ U/mL; Nb.BtsI, 56.4 U/mL; DFHBI, 8 μM; NTPs, 4 mM each): (i) 0.1 μM, (ii) 0.2 μM, and (iii) 0.4 μM. (G) Transient time-dependent catalytic rates corresponding to the transcription of the DFHBI-aptamer in the presence of different concentrations of L_2_: (i) 0.1 μM, (ii) 0.2 μM, and (iii) 0.4 μM. (H) Temporal fluorescence changes upon cyclic stepwise operation of the nickase-stimulated operation of the transcription of the DFHBI-aptamer by adding the fuel strand L_2_: 0.04 μM and 0.2 μM (T_7_/A_5_, 0.04 μM; T7 RNAP, 1.25 × 10^3^ U/mL; Nb.BtsI, 56.4 U/mL; DFHBI, 8 μM; NTPs, 4 mM each). (Arrow indicates time-interval use the trigger L_2_ activates the second transient transcription cycle.) (I) Time-dependent catalytic rates upon stepwise operation of the transcription cycles of the DFHBI-RNA aptamer.

A third mechanism to regulate the operation of transcription templates using a fuel-strand as activator and a nickase as catalyst dissipating the active structure of the template is introduced to stimulate transient transcription machineries and gated, transient transcription machineries. Figure 5(A) presents the reaction module consisting of the inactive template T_6_/A_4_, the mixture NTPs, the T7 RNAP, and the nicking enzyme Nt.BbvCI. Subjecting the reaction module to the fuel strand L_1_, results in its hybridization with a part of the free single-strand tether T_6_. Hybridization of L_1_ with the template completes the promoter-activated machinery, resulting in transcription of the MG-RNA aptamer. The strand L_1_ was encoded, however, to include the sequence-specific domain to be nicked by Nt.BbvCI. Cleavage of L_1_ leads to “waste” fragments, being separated from the transcription template, resulting in the blockage of the transcription process and the recovery of the inactive reaction module. This dynamic set of reactions leads to the transient temporal formation of the MG-RNA aptamer, a process that is followed by the transient evolution of the fluorescent MG aptamer complex. Figure 5(B) depicts the temporal fluorescence changes of the MG aptamer complex generated in the presence of different concentrations of fuel strand L_1_. The fluorescence intensities are higher as the concentrations of L_1_ increase. These results are consistent with the enrichment of the active transcription template upon elevating the concentration of L_1_ and the temporal blockage of the transcription machinery by the nickase-stimulated depletion of the active transcription template, leading to a constant accumulation of MG-RNA aptamer. (For the formation of a kinetic model and the simulation of the temporal fluorescence changes of the nickase-driven transient transcription of the MG-RNA aptamer, in the presence of different fuel concentrations, see Figures S21 and S22 and Table S3 in the Supporting Information and accompanying discussion.) The temporal fluorescence changes of the MG aptamer depicted in Figure 5(B) were translated into time-dependent catalytic rates corresponding to the formation of the MG aptamer in the presence of different concentrations of L_1_, and these are displayed in Figure 5(C). The catalytic rates reveal transient behavior of the transcription and increase in the catalytic rate generating the MG aptamer for a time duration of ca. 20 min followed by a time-dependent decay due to the temporal nickase-stimulated deactivation of the dynamic process that fully depletes the active transcription template after ca. 60 min. (For duplicate experimental results of nickase-driven transcription machinery, see Figure S23. For a control experiment of nickase-driven transcription machinery demonstrating the inhibited lack of leakage of the transcription machinery, in the absence of the fuel strand L_1_, see Figure S24. For additional experiments probing the nickase-guided transient operation of the transcription machinery in the presence of variable concentrations of NTPs and nickase, see Figures S25 and S26.) The reaction module can be reactivated by re-addition of the fuel strand L_1_, Figure 5(D) and (E). Similarly, a reaction module for the transient transcription of the DFHBI-RNA aptamer was assembled using template T_7_/A_5_ and the nickase Nb.BtsI (see Figure S27). Figure 5(F) depicts the temporal fluorescence changes corresponding to the dynamic transcription machinery in the presence of variable concentrations of trigger L_2_. As the concentration of L_2_ increases, the fluorescence changes in the DFHBI aptamer are higher. Figure 5(G) shows the translation of the temporal fluorescence changes shown in Figure 5(F) into time-dependent catalytic rates corresponding to the formation of the DFHBI-aptamer guided by the nickase constituent. Figure 5(H) and (I) demonstrate the cyclic reactivation of the transcription machinery related to the synthesis of DFHBI-aptamer by re-addition of the fuel strand L_2_ (time marked with arrow).

**Figure 6:**
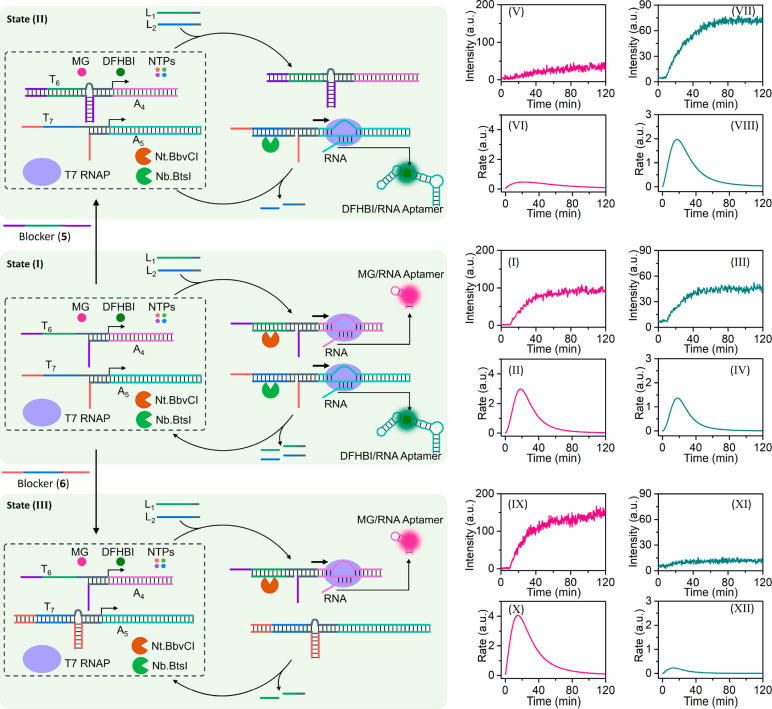
Schematic configuration of a reaction module in State I, consisting of two templates being triggered by fuel strands (L_1_, L_2_) and T7 RNAP/NTPs to operate transcription machineries being transiently modulated by two nickases (Nt.BbvCI and Nb.BtsI) to transcribe two RNA aptamers. Panels I and II show temporal fluorescence changes and time-dependent catalytic rates corresponding to the transiently modulated transcription of the MG-RNA aptamer. Panels III and IV show temporal fluorescence changes and catalytic rates corresponding to the transiently modulated transcription of the DFHBI-RNA aptamer (T_6_/A_4_, 0.02 μM; T_7_/A_5_, 0.06 μM; T7 RNAP, 1.25 × 10^3^ U/mL; Nt.BbvCI, 123.9 U/mL; Nb.BtsI, 94 U/mL; MG, 4 μM; DFHBI, 8 μM; NTPs, 4 mM each; L_1_, 0.25 μM; L_2_, 0.45 μM). Subjecting State I to blocker **5** (0.4 μM) yields State 2, where template T_6_/A_4_ is inhibited, leading to the gated transcription of the DFHBI-RNA aptamer. Panels V and VI demonstrate the inhibition of the transcription of the MG-RNA aptamer, whereas Panels VII and VIII present the temporal fluorescence changes and catalytic rates corresponding to the gated transcription of the DFHBI-RNA aptamer. Subjecting State I to blocker **6** (1.2 μM), yielding State III, leads to the inhibition of the template T_7_/A_5_ and to the gated nickase modulated transient transcription of the MG-RNA aptamer. Panels IX and X show the temporal fluorescence changes and catalytic rates associated with the gated and selective transcription of the MG-RNA aptamer, while the transcription of the DFHBI-RNA aptamer is inhibited, Panels XI/XII.

Moreover, as before, the nickase-stimulated transient transcription machinery was further engineered to drive gated transient machineries in the presence of two different nickases and appropriate blocker units, as shown in Figure 6. The reaction module consists of two templates, T_6_/A_4_ and T_7_/A_5_, two sequence-specific nickases, Nt.BbvCI and Nb.BtsI, T7 RNAP, the NTPs, and the ligands MG and DFHBI. Subjecting the reaction module to the triggering strands L_1_ and L_2_ activates the two templates, resulting in the operation of the two transcription machineries and the formation of the MG-RNA aptamer and DFHBI-RNA aptamer. The concomitant nicking of the strands L_1_ and L_2_ by the Nt.BbvCI and Nb.BtsI nickases degrades the respective templates, leading to the transient transcription of the two RNA aptamers and the recovery of the parent inactive reaction module. Figure 6, Panels I/II and III/IV, depicts the temporal fluorescence changes upon formation of the MG-RNA aptamer and the DFHBI-RNA aptamer and the transient catalytic rates generating the two aptamers. Treatment of the reaction module with the blocker strand **5** blocks the template T_6_/A_4_, prohibiting the activation of the template T_6_/A_4_ by L_1_, State II. As a result, in the presence of the triggers L_1_ and L_2_, the gated activation of template T_7_/A_5_ leads to the selective transcription of the DFHBI-RNA only. Figure 6, Panels V/VI and VII/VIII, reveals the gated temporal transcription of the DFHBI-RNA aptamer and the blockage of the transcription of the MG-RNA aptamer. Note that, the temporal fluorescence changes of the DFHBI in State II are substantially higher than the temporal fluorescence intensities of the DFHBI aptamer in State I. This enhanced transcription of the DFHBI-aptamer in State II is consistent with the fact that blockage of the competitive MG-aptamer transcription guides the active concentrations of the NTPs/T7 RNAP available in the reaction module toward the transcription of the DFHBI-RNA aptamer. Similarly, subjecting the reaction module in State I to the blocker strand **6** yields State III, where temporal T_7_/A_5_ is blocked. This leads, in the presence of L_1_ and L_2_, to the selective operation of the template T_6_/A_4_, resulting in the temporal, transient transcription of the MG-RNA aptamer, Figure 6, Panels IX/X and XI/XII, respectively. As before, the temporal fluorescence changes upon the gated transcription of the MG-RNA aptamer are enhanced, as compared to the formation of the MG aptamer in State I, due to the blockage of the competitive transcription path of the DFHBI-RNA aptamer.

**Figure 7:**
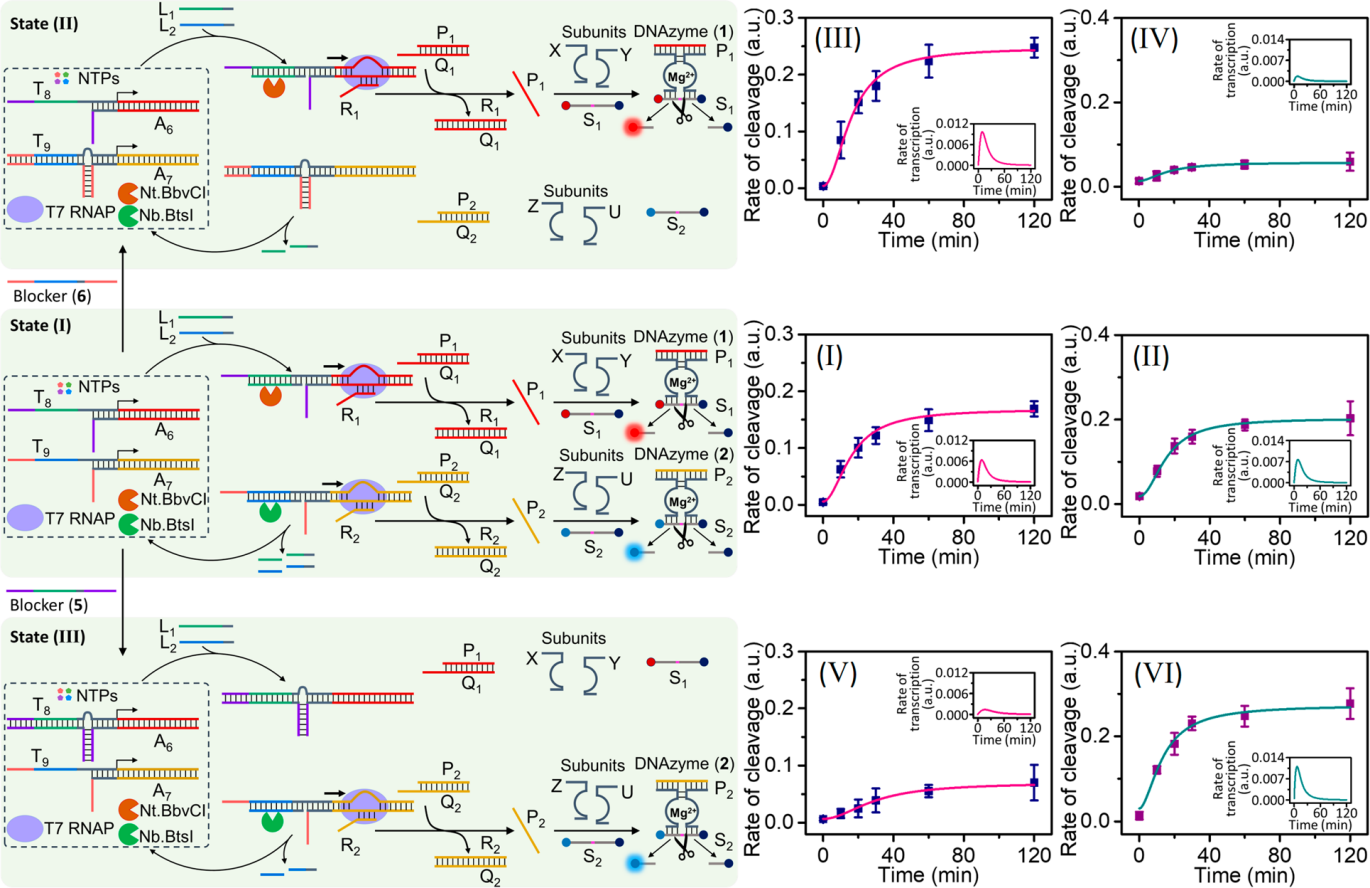
Schematic configuration of a reaction module in State I, leading to the gated transiently modulated transcription machinery guiding the transient operation of two DNAzyme catalysts. The transient modulated transcription machinery applies two nickases as auxiliary catalysts, controlling the transient transcription of strands R_1_ and R_2_. The resulting strands interact (displace) auxiliary modules P_1_/Q_1_ and P_2_/Q_2_ to yield temporal operating DNAzyme **1** and DNAzyme **2**. Panels I and II depict the temporal rates of cleavage of substrate S_1_ (by DNAzyme **1**) and substrate S_2_ (by DNAzyme **2**) (T_8_/A_6_, 0.02 μM; T_9_/A_7_, 0.08 μM; T7 RNAP, 1.67 × 10^3^ U/mL; Nt.BbvCI, 166.7 U/mL; Nb.BtsI, 133.3 U/mL; NTPs, 4 mM each; L_1_, 0.2 μM; L_2_, 0.64 μM; P_1_/Q_1_, 1 μM; P_2_/Q_2_, 1 μM; X, Y, Z, and U, 1 μM each; S_1_ and S_2_, 2 μM each). Insets in Panels I and II present the time-dependent rates of formation of R_1_ and R_2_ by the modulated transient transcription machineries. Subjecting State I to blocker **6** (1.6 μM) results in the reaction module in State II, that results in the gated transiently modulated transcription of R_1_ and the guided selective formation of DNAzyme **1**. Panel III shows the temporal rates of cleavage of S_1_ by DNAzyme **1**, and the inset presents the transiently modulated transcription rate of R_1_. Panel IV demonstrates the inhibition of DNAzyme **2** upon operation of the reaction module in State II. Treatment of State I with blocker **5** (0.4 μM) results in the reaction module in State III, that leads to the gated transiently modulated transcription of R_2_ and the guided selective formation of DNAzyme **2**. Panel V demonstrates the inhibition of DNAzyme **1** in State III. Panel VI shows the temporal rates of cleavage of S_2_ by DNAzyme **2**, and the inset presents the transiently operated transcription rate of R_2_. (For the raw data corresponding to the respective gated DNAzyme activities, see Figures S28–S30.)

Following the development of three alternative methods driving transient transcription machineries and gated transient transcription systems, we aimed to demonstrate the possible utility of these dynamic transient transcription networks, particularly toward the assembly of transcription-guided catalytic processes. This is exemplified in Figure 7 with the application of the gated nickase-driven transcription machinery for the transient operation of two different programmed Mg^2+^-ion-dependent DNAzymes. The reaction module, State I, consists of two inactive transcription templates T_8_/A_6_ and T_9_/A_7_, the T7 RNAP, NTPs, and the two nicking enzymes, Nt.BbvCI and Nb.BtsI. In the presence of the triggering strands L_1_ and L_2_, the two transcription templates are activated to yield the transcription products R_1_ and R_2_. The concomitant nicking of the triggering strands L_1_ and L_2_, associated with the templates by the two nickases, Nt.BbvCI and Nb.BtsI, depletes the active transcription templates and drives the recovery of the parent, rest reaction module, leading to the transient and temporal transcription of the products R_1_ and R_2_. The latter products act, however, as precursors for the transient operation of two different Mg^2+^-ion-dependent DNAzymes. Extrusion of samples at time intervals of the operating temporal, transient transcription of the products R_1_ and R_2_ into a mixture consisting of the duplexes P_1_/Q_1_ and P_2_/Q_2_ and the DNAzyme subunits X, Y and Z, U and the respective fluorophore/quencher- and ribonucleobase-modified DNAzyme substrates, S_1_ and S_2_, results in a sequence of processes generating the temporal formation of two Mg^2+^-ion-dependent DNAzymes, DNAzyme **1** and DNAzyme **2**, and to the temporal, transient cleavage of the substrates S_1_ and S_2_. The transcription products R_1_ and R_2_ displace the duplexes P_1_/Q_1_ and P_2_/Q_2_ to yield the duplexes R_1_/Q_1_ and R_2_/Q_2_, and the released strands P_1_ and P_2_ are pre-engineered to assemble the DNAzyme **1** and DNAzyme **2**. The two assembled DNAzymes cleave the respective fluorophore/quencher-labeled substrates (FAM/IBRQ for S_1_ and Cy5/BHQ2 for S_2_), and the resulting temporal fluorescence changes probe the temporal activities of DNAzyme **1** and DNAzyme **2**. Note that the two Mg^2+^-ion-dependent DNAzymes **1** and **2** differ in the sequence of the binding arms, leading to the specific cleavage of the two different substrates S_1_, S_2_. Moreover, because the transcription machineries guide the temporal transient formation of R_1_ and R_2_, the dynamic features of the transcribed products are translated into dynamic catalytic features of the resulting DNAzymes. That is, the DNAzymes reveal transient catalytic activities. Figure 7, Panels I and II, depicts the temporal catalytic rates corresponding to fluorescence changes associated with the temporal cleavage of the substrates S_1_ and S_2_, respectively. The insets in Panels I and II present the derived curves of the transient operation of catalytic rates of the DNAzymes, imaging the parent transient transcription rates generating R_1_ and R_2_. Subjecting the reaction module in State I to the blocker unit **6** inhibits the transcription template T_9_/A_7_, leading to the gated operation of the template T_8_/A_6_, State II. This leads to the transient formation of R_1_ and the secondary gated operation of DNAzyme **1**, cleaving S_1_. Panels III and IV show the temporal catalytic rates of the DNAzymes upon operating the **6**-inhibited reaction module in State II, demonstrating the effective temporal catalytic rates of DNAzyme **1** and the blocked catalysis of DNAzyme **2**. Similarly, treatment of the reaction module in State I with the blocker unit **5** leads to the gated inhibition of the template T_8_/A_6_, State III. Under these conditions, template T_9_/A_7_ is activated toward the transient transcription of R_2_ and the subsequent R_2_-guided operation of DNAzyme **2**. Panels V and VI show the temporal catalytic rates upon the **5**-inhibited operation of the gated reaction module in State III. While DNAzyme **2** reveals temporal cleavage of the substrate S_2_ and transient catalytic rates, guided by R_2_, DNAzyme **1** is inhibited.

The study introduced three alternative methods to trigger transient machineries. The results raise, however, fundamental questions related to possible advantages/disadvantages associated with the different methods and possible benefits of having three alternative triggered transient transcription machineries. Upon comparing the nickase-and DNAzyme-modulated transient machineries to the strand-displacement-modulated machinery, we may realize that the latter is less economic due to the parallel consumption of the NTPs bases. Moreover, the nickase-modulated triggered transcription machinery requires an additional enzyme to the T7 RNAP, adding disadvantages associated with the stability of the nickase and eventually the need to adjust auxiliary system conditions such as temperature or buffer composition. Furthermore, the availability of three alternative triggering modes to stimulate the transcription machineries introduces further possibilities to enhance the complexity of the systems by integrating several of the transcription machinery modes into a single system operating in parallel. Under such conditions, the integration of different transcription templates, different respective triggering fuels, and engineering of the templates to yield different RNA output products, while ensuring lack of destructive inhibition or crosstalks between the system constituents, is essential. This is exemplified in Figures S31–S33, with the parallel operation of two transient transcription machineries using the DNAzyme-modulated, strand-displacement-modulated, and nickase-driven transcription machineries transcribing the MG-RNA aptamer and the DFHBI-RNA aptamer, respectively.

## Conclusions

The present study has introduced three alternate methods to operate transient transcription machineries and gated transient transcription machineries—the design of reaction modules that include the dynamic DNAzyme-, strand-displacement-, or nickase-stimulated dissipation of the active transcription templates, leading to the transient activities of the systems. All three methods were used to operate the gated transient synthesis of two different RNA aptamers by integrating two alternative transcription templates into the reaction modules and dictating the operation of the desired machinery by applying appropriate blockers. The significance of these methods rests, however, on diversity and enhanced complexity capacities to operate multimode transcription machineries synthesizing selective mixtures of RNAs. The reaction modules could include any number of transcription machineries and modulating blocker units that control the number and composition of evolved RNAs using appropriate regulators. Furthermore, by the integration more than one regulator, e.g., different strand displacers or different nickases, the complexities of the systems might be even further enhanced.

At present, the different transiently modulated transcription circuits are operating in homogeneous buffer solutions. Recent efforts are directed, however, to integrate triggered transcription machineries into cell-like containments, such as hydrogel microcapsules^58^ or liposomes.^57^ The integration of transiently modulated transcription machineries into such cell-mimicking microenvironments might be an important step in future “protocell” design.^54,56^

## Experimental Section

**Chart 1:**
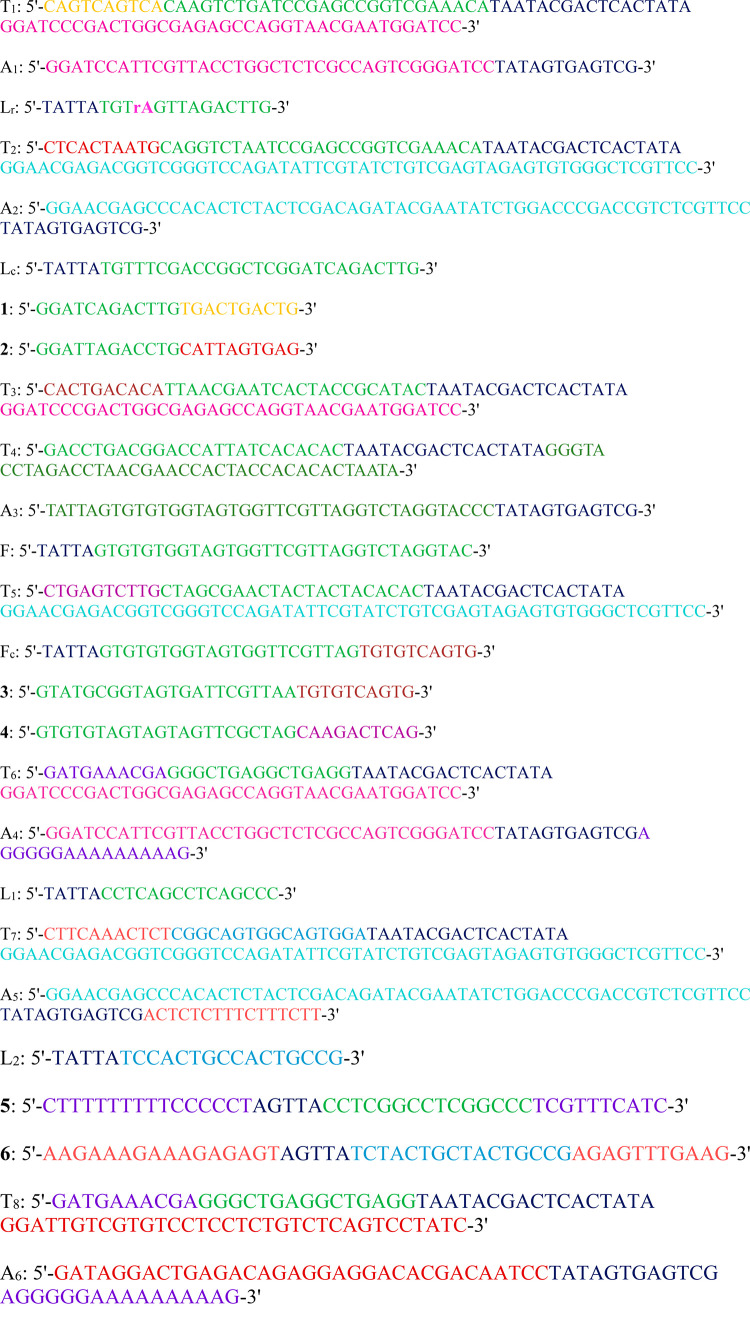

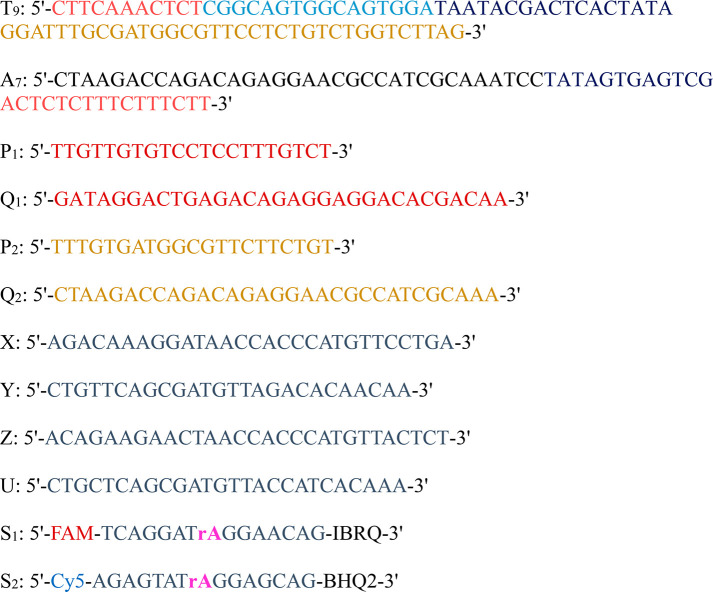


Oligonucleotides were purchased from Integrated DNA Technologies and Sigma-Aldrich. The sequences of the oligonucleotides are listed in Chart 1.

Detailed procedures to prepare and perform the different transient transcription systems and to characterize the systems are presented in the Supporting Information.

## Supporting Information Available

Details describing the enzymes, chemicals, composition of the transient and gated reaction modules transcribing the MG/DFHBI aptamers, methods to follow real-time fluorescence changes of the MG/DFHBI-RNA aptamer complexes, dynamic and gated formation of the DNAzymes and their activities, and control experiments associated with the transient transcriptional systems (PDF)
