# Supplementary material for: Alternate Strategies to Induce Dynamically Modulated Transient Transcription Machineries
Source: ACS Nano. 2023 Sep 5;17(18):18266–79. doi: 10.1021/acsnano.3c05336 (PMC10540262; doi:10.1021/acsnano.3c05336)
Supplement: Supplementary file 1 — nn3c05336_si_001.pdf [file nn3c05336_si_001.pdf]

Supporting Information

Alternate Strategies to Induce Dynamically Modulated Transient Transcription Machineries

Zhenzhen Li‡, Jianbang Wang‡, Itamar Willner*

The Institute of Chemistry, The Center for Nanoscience and Nanotechnology, The Hebrew University of Jerusalem, Jerusalem 91904, Israel.

‡These authors contributed equally to this study.

*E-mail: Itamar.willner@mail.huji.ac.il

Materials

All the enzymes, including T7 RNAP (50,000 U/mL), Nt.BbvCI (10,000 U/mL) and Nb.BtsI (10,000 U/mL), RNAP reaction buffer, and ribonucleotide solution mix (rATP, rCTP, rGTP, and rUTP) were purchased from New England Biolabs Inc. MG (Malachite Green) and DFHBI ((5Z)-5-[(3,5-difluoro-4-hydroxyphenyl)methylene]-3,5-dihydro-2,3-dimethyl-4*H*-imidazol-4-one) were purchased from Sigma-Aldrich. Oligonucleotides were purchased from Integrated DNA Technologies and Sigma-Aldrich. All the sequences of the oligonucleotides were listed below:

T_1: 5'-CAGTCAGTCACAAGTCTGATCCGAGCCGGTCGAAACATAATACGACTCACTATAGGATCCCGACTGGCGAGAGCCAGGTAACGAATGGATCC-3'

A_1: 5'-GGATCCATTCGTTACCTGGCTCTCGCCAGTCGGGATCCTATAGTGAGTCG-3'

L_r: 5'-TATTATGT**rA**GTTAGACTTG-3'

T_2: 5'-CTCACTAATGCAGGTCTAATCCGAGCCGGTCGAAACATAATACGACTCACTATAGGAACGAGACGGTCGGGTCCAGATATTCGTATCTGTCGAGTAGAGTGTGGGCTCGTTCC-3'

A_2: 5'-GGAACGAGCCCACACTCTACTCGACAGATACGAATATCTGGACCCGACCGTCTCGTTCCTATAGTGAGTCG-3'

L_c: 5'-TATTATGTTTCGACCGGCTCGGATCAGACTTG-3'

(**1**): 5'-GGATCAGACTTGTGACTGACTG-3'

(**2**): 5'-GGATTAGACCTGCATTAGTGAG-3'

T_3: 5'-CACTGACACATTAACGAATCACTACCGCATACTAATACGACTCACTATAGGATCCCGACTGGCGAGAGCCAGGTAACGAATGGATCC-3'

T_4: 5'-GACCTGACGGACCATTATCACACACTAATACGACTCACTATAGGGTACCTAGACCTAACGAACCACTACCACACACTAATA-3'

A_3: 5'-TATTAGTGTGTGGTAGTGGTTCGTTAGGTCTAGGTACCCTATAGTGAGTCG-3'

F: 5'-TATTAGTGTGTGGTAGTGGTTCGTTAGGTCTAGGTAC-3'

T_5: 5'-CTGAGTCTTGCTAGCGAACTACTACTACACACTAATACGACTCACTATAGGAACGAGACGGTCGGGTCCAGATATTCGTATCTGTCGAGTAGAGTGTGGGCTCGTTCC-3'

Fc: 5'-TATTAGTGTGTGGTAGTGGTTCGTTAGTGTGTCAGTG-3'

(**3**): 5'-GTATGCGGTAGTGATTCGTTAATGTGTCAGTG-3'

(**4**): 5'-GTGTGTAGTAGTAGTTCGCTAGCAAGACTCAG-3'

T_6: 5'-GATGAAACGAGGGCTGAGGCTGAGGTAATACGACTCACTATAGGATCCCGACTG GCGAGAGCCAGGTAACGAATGGATCC-3'

A_4: 5'-GGATCCATTCGTTACCTGGCTCTCGCCAGTCGGGATCCTATAGTGAGTCGAGGGG GAAAAAAAAAG-3'

L_1: 5'-TATTACCTCAGCCTCAGCCC-3'

T_7: 5'-CTTCAAACTCTCGGCAGTGGCAGTGGATAATACGACTCACTATAGGAACGAGACG GTCGGGTCCAGATATTCGTATCTGTCGAGTAGAGTGTGGGCTCGTTCC-3'

A_5: 5'-GGAACGAGCCCACACTCTACTCGACAGATACGAATATCTGGACCCGACCGTCTC GTTCCTATAGTGAGTCGACTCTCTTTCTTTCTT-3'

L_2: 5'-TATTATCCACTGCCACTGCCG-3'

(**5**): 5'-CTTTTTTTTTCCCCCTAGTTACCTCGGCCTCGGCCCTCGTTTCATC-3'

(**6**): 5'-AAGAAAGAAAGAGAGTAGTTATCTACTGCTACTGCCGAGAGTTTGAAG-3'

T_8: 5'-GATGAAACGAGGGCTGAGGCTGAGGTAATACGACTCACTATAGGATTGTCGTGTC CTCCTCTGTCTCAGTCCTATC-3'

A_6: 5'-GATAGGACTGAGACAGAGGAGGACACGACAATCCTATAGTGAGTCGAGGGGGA AAAAAAAAG-3'

T_9: 5'-CTTCAAACTCTCGGCAGTGGCAGTGGATAATACGACTCACTATAGGATTTGCGAT GGCGTTCCTCTGTCTGGTCTTAG-3'

A_7: 5'-CTAAGACCAGACAGAGGAACGCCATCGCAAATCCTATAGTGAGTCGACTCTCTTT CTTTCTT-3'

P_1: 5'-TTGTTGTGTCCTCCTTTGTCT-3'

Q_1: 5'-GATAGGACTGAGACAGAGGAGGACACGACAA-3'

P_2: 5'-TTTGTGATGGCGTTCTTCTGT-3'

Q_2: 5'-CTAAGACCAGACAGAGGAACGCCATCGCAAA-3'

X: 5'-AGACAAAGGATAACCACCCATGTTCCTGA-3'

Y: 5'-CTGTTCAGCGATGTTAGACACAACAA-3'

Z: 5'-ACAGAAGAACTAACCACCCATGTTACTCT-3'

U: 5'-CTGCTCAGCGATGTTACCATCACAAA-3'

S_1: 5'-FAM-TCAGGAT**rA**GGAACAG-IBRQ-3'

S_2: 5'-Cy5-AGAGTAT**rA**GGAGCAG-BHQ2-3'

Methods

Dynamic transcription of the MG aptamer from the Pb^{2+}-ion-dependent DNAzyme driven transient transcription machinery

The transient reaction module for the transcription of the MG aptamer was a mixture of the DNA template T_1/A_1 (0.1 μM), T7 RNAP (1.25 × 10^3 U/mL), MG (4 μM), the NTPs (4 mM each) and Pb^{2+} ions (0.1 μM) in the RNAP reaction buffer (40 mM Tris-HCl, 1 mM DTT, 2 mM spermidine, 20 mM $MgCl_2$, pH 7.9). Variable concentrations of the fuel strand L_r (0.05 μM, 0.1 μM and 0.15 μM) were applied to the reaction module and the dynamic time-dependent fluorescence changes of the MG-aptamer complex were recorded with a Cary Eclipse Fluorometer (Varian Inc.) at λ_{em} = 665 nm (30 °C). For duplicate experimental results of Pb^{2+}-DNAzyme-modulated transcription machinery, see Figure S4. The time-dependent fluorescence changes were translated into temporal catalytic rates by primary smoothing of the experimental curves following by taking the derivative of the fitted curve.

For the cyclic transient transcription of the MG aptamer, the reaction module was applied twice with the fuel strand L_r: 0.1 μM and 0.5 μM. The dynamic time-dependent fluorescence changes of the MG-aptamer complex were recorded with a Cary Eclipse Fluorometer (Varian Inc.) at λ_{em} = 665 nm (30 °C).

For the measurement of the dynamic transcription of the MG aptamer at variable concentrations of NTPs, three reaction module samples were prepared including the same concentrations of DNA template T_1/A_1 (0.05 μM), T7 RNAP (1.25 × 10^3 U/mL) and MG (4 μM), and different concentrations of the NTPs (2 mM, 4 mM and 6 mM, respectively). All the three samples were applied with the same concentrations of the fuel strand L_r (0.1 μM) and the time-dependent fluorescence changes were recorded with a Cary Eclipse Fluorometer (Varian Inc.) at λ_{em} = 665 nm (30 °C).

For the control experiment, the strand L_c was used as the trigger. The reaction mixture included the DNA template T_1/A_1 (0.05 μM), T7 RNAP (1.25 × 10^3 U/mL), MG (4 μM) and the NTPs (4 mM each). Strand L_c (0.1 μM) was applied to the reaction module and the time-dependent fluorescence changes were recorded with a Cary Eclipse Fluorometer (Varian Inc.) at λ_{em} = 665 nm

(30 °C).

Dynamic transcription of the DFHBI aptamer from the Pb^{2+}-ion-dependent DNAzyme driven transient transcription machinery

The transient reaction module for the transcription of the DFHBI aptamer was a mixture of the DNA template T_2/A_2 (0.2 μM), T7 RNAP (1.875 × 10^3 U/mL), DFHBI (8 μM), the NTPs (4 mM each) and the Pb^{2+} ions (0.1 μM) in the RNAP reaction buffer (40 mM Tris-HCl, 1 mM DTT, 2 mM spermidine, 20 mM $MgCl_2$, pH 7.9). Variable concentrations of the fuel strand L_r (0.1 μM, 0.2 μM and 0.3 μM) were applied to the reaction module and the dynamic time-dependent fluorescence changes of the DFHBI-aptamer complex were recorded with a Cary Eclipse Fluorometer (Varian Inc.) at λ_{em} = 500 nm (30 °C).

For the cyclic transient transcription of the DFHBI aptamer, the reaction module including the DNA template T_2/A_2 (0.1 μM) was applied twice with the fuel strand L_r: 0.1 μM and 0.2 μM. The dynamic time-dependent fluorescence changes of the DFHBI-aptamer complex were recorded with a Cary Eclipse Fluorometer (Varian Inc.) at λ_{em} = 500 nm (30 °C).

Transient Gated operations of the Pb^{2+}-ion-dependent DNAzyme driven transcription machinery

The mixture of the reaction module included two DNA templates, T_1/A_1 (0.05 μM) and T_2/A_2 (0.2 μM), and the T7 RNAP (1.25 × 10^3 U/mL), MG (4 μM), DFHBI (8 μM) and NTPs (4 μM each) and the Pb^{2+} ions (0.1 μM) in the RNAP reaction buffer (40 mM Tris-HCl, 1 mM DTT, 2 mM spermidine, 20 mM $MgCl_2$, pH 7.9). The transient transcription of the templates were triggered by adding the fuel strand L_r, 0.1 μM. The dynamic transcription of the MG and DFHBI RNA aptamers were followed by the time-dependent fluorescence changes of the MG-aptamer complex at λ_{em} = 665 nm and of the DFHBI-aptamer complex at λ_{em} = 500 nm, recording with a Cary Eclipse Fluorometer (Varian Inc.) at 30 °C.

To gate the respective transcription processes, the reaction module was subjected to the blocker (**1**) (2 μM) or (**2**) (4 μM), for a time interval of 10 min, and the respective dynamic transcription processes were triggered by L_r, 0.1 μM, followed by monitoring the time-dependent fluorescence changes of the respective RNA aptamer-ligand complexes using a Cary Eclipse Fluorometer (Varian

Inc.) at 30 °C.

Dynamic transcription of the MG aptamer from the strand-displacement guided transient transcription machinery

The transient reaction module for the transcription of the MG aptamer was a mixture of the DNA templates T_3/A_1 (0.05 μM) and T_4/A_3 (0.05 μM), T7 RNAP (2.5×10^3 U/mL), MG (4 μM), the NTPs (4 mM each) in the RNAP reaction buffer (40 mM Tris-HCl, 1 mM DTT, 2 mM spermidine, 20 mM $MgCl_2$, pH 7.9). Variable concentrations of the fuel strand F (0.05 μM, 0.1 μM and 0.15 μM) were applied to the reaction module and the dynamic time-dependent fluorescence changes of the MG-aptamer complex were recorded with a Cary Eclipse Fluorometer (Varian Inc.) at λ_{em} = 665 nm (37 °C). For duplicate experimental results of strand-displacement-stimulated transcription machinery, see Figure S11.

For the cyclic transient transcription of the MG aptamer, the reaction module was applied twice with the fuel strand F: 0.033 μM and 0.33 μM. The dynamic time-dependent fluorescence changes of the MG-aptamer complex were recorded with a Cary Eclipse Fluorometer (Varian Inc.) at λ_{em} = 665 nm (37 °C).

For the measurement of the dynamic transcription of the MG aptamer at variable concentrations of NTPs, three reaction module samples were prepared including the same concentrations of DNA templates, T_3/A_1 (0.05 μM) and T_4/A_3 (0.05 μM), T7 RNAP (1.875×10^3 U/mL) and MG (4 μM), and different concentrations of the NTPs (1 mM, 2 mM and 4 mM, respectively). All the three samples were applied with the same concentrations of the fuel strand F, 0.1 μM and the time-dependent fluorescence changes were recorded with a Cary Eclipse Fluorometer (Varian Inc.) at λ_{em} = 665 nm (37 °C).

For the measurement of the dynamic transcription of the MG aptamer at variable concentrations of DNA template T_4/A_3, three reaction module samples were prepared including the same concentrations of DNA template, T_3/A_1 (0.05 μM), T7 RNAP (2.5×10^3 U/mL) and MG (4 μM), and different concentrations of the DNA template T_4/A_3 (0.1 μM, 0.05 μM and 0.02 μM, respectively). All the three samples were applied with the same concentrations of the fuel strand F, 0.15 μM and the time-dependent fluorescence changes were recorded with a Cary Eclipse

Fluorometer (Varian Inc.) at λ_{em} = 665 nm (37 °C).

For the control experiment, the strand Fc was used as the trigger. The reaction mixture included the DNA templates, T_3/A_1 (0.05 μM) and T_4/A_3 (0.05 μM), T7 RNAP (1.875 × 10^3 U/mL), MG (4 μM) and the NTPs (4 mM each). Strand Fc (0.1 μM) was applied to the reaction module and the time-dependent fluorescence changes were recorded with a Cary Eclipse Fluorometer (Varian Inc.) at λ_{em} = 665 nm (37 °C).

For the polyacrylamide gel electrophoresis (PAGE) measurement, three samples were prepared including F (2 μM, 10 μL), T_4+F/A_3 (0.5 μM, 10 μL) and T_4+F/A_3 (0.5 μM) + F (2 μM) (10 μL). All the samples were loaded into the 10% PAGE gel to perform at 80 V for 16 h (8 °C).

Dynamic transcription of the DFHBI aptamer from the strand-displacement guided transient transcription machinery

The transient reaction module for the transcription of the DFHBI aptamer was a mixture of the DNA templates T_5/A_2 (0.05 μM) and T_4/A_3 (0.02 μM), T7 RNAP (2.5 × 10^3 U/mL), DFHBI (8 μM), the NTPs (4 mM each) in the RNAP reaction buffer (40 mM Tris-HCl, 1 mM DTT, 2 mM spermidine, 20 mM $MgCl_2$, pH 7.9). Variable concentrations of the fuel strand F (0.05 μM, 0.1 μM and 0.15 μM) were applied to the reaction module and the dynamic time-dependent fluorescence changes of the DFHBI-aptamer complex were recorded with a Cary Eclipse Fluorometer (Varian Inc.) at λ_{em} = 500 nm (37 °C).

For the cyclic transient transcription of the DFHBI aptamer, the reaction module including the DNA templates T_5/A_2 (0.05 μM) and T_4/A_3 (0.05 μM), T7 RNAP (2.5 × 10^3 U/mL), DFHBI (8 μM), and the NTPs (4 mM each) was applied twice with the fuel strand F: 0.05 μM and 0.15 μM. The dynamic time-dependent fluorescence changes of the DFHBI-aptamer complex were recorded with a Cary Eclipse Fluorometer (Varian Inc.) at λ_{em} = 500 nm (37 °C).

Transient Gated operations of the strand-displacement guided transient transcription machinery

The mixture of the reaction module contained three DNA templates, T_3/A_1 (0.02 μM), T_4/A_3 (0.03 μM) and T_5/A_2 (0.06 μM), and the T7 RNAP (2.06 × 10^3 U/mL), MG (4 μM), DFHBI (8 μM) and NTPs (4 mM each) in the RNAP reaction buffer (40 mM Tris-HCl, 1 mM DTT, 2 mM spermidine,

20 mM $MgCl_2$, pH 7.9). The transient transcription of the templates were triggered by adding the fuel strand F, 0.03 μM. The dynamic transcription of the MG and DFHBI aptamers were followed by the time-dependent fluorescence changes of the MG-aptamer complex at λ_{em} = 665 nm and of the DFHBI-aptamer complex at λ_{em} = 500 nm, recording with a Cary Eclipse Fluorometer (Varian Inc.) at 37 °C.

To gate the respective transcription processes, the reaction module was subjected to the blocker (**3**) (0.2 μM) or (**4**) (0.3 μM), for a time interval of 10 min, and the respective dynamic transcription processes were triggered by F, 0.03 μM, followed by monitoring the time-dependent fluorescence changes of the respective RNA aptamer-ligand complexes using a Cary Eclipse Fluorometer (Varian Inc.) at 37 °C.

Dynamic transcription of the MG aptamer from the nickase guided transient transcription machinery

The transient reaction module for the transcription of the MG aptamer was a mixture of the DNA template T_6/A_4 (0.02 μM), T7 RNAP (1.25×10^3 U/mL), Nt.BbvCI (83.3 U/mL), MG (4 μM), the NTPs (4 mM each) in the RNAP reaction buffer (40 mM Tris-HCl, 1 mM DTT, 2 mM spermidine, 20 mM $MgCl_2$, pH 7.9). Variable concentrations of the fuel strand L_1 (0.1 μM, 0.2 μM and 0.3 μM) were applied to the reaction module and the dynamic time-dependent fluorescence changes of the MG-aptamer complex were recorded with a Cary Eclipse Fluorometer (Varian Inc.) at λ_{em} = 665 nm (37 °C). For duplicate experimental results of nickase-driven transcription machinery, see Figure S23.

For the cyclic transient transcription of the MG aptamer, the reaction module was applied twice with the fuel strand L_1: 0.02 μM and 0.1 μM. The dynamic time-dependent fluorescence changes of the MG-aptamer complex were recorded with a Cary Eclipse Fluorometer (Varian Inc.) at λ_{em} = 665 nm (37 °C).

For the measurement of the dynamic transcription of the MG aptamer at variable concentrations of NTPs, three reaction module samples were prepared including the same concentrations of DNA template, T_6/A_4 (0.02 μM), T7 RNAP (1.25×10^3 U/mL), Nt.BbvCI (87.5 U/mL), and MG (4 μM), and different concentrations of the NTPs (1.33 mM, 2.66 mM and 4 mM, respectively). All the three

samples were applied with the same concentrations of the fuel strand L_1, 0.1 μM and the time-dependent fluorescence changes were recorded with a Cary Eclipse Fluorometer (Varian Inc.) at λ_{em} = 665 nm (37 °C).

For the measurement of the dynamic transcription of the MG aptamer at variable concentrations of Nt.BbvCI, three reaction module samples were prepared including the same concentrations of DNA template, T_6/A_4 (0.02 μM), T7 RNAP (1.25 × 10^3 U/mL) and MG (4 μM), and different concentrations of the Nt.BbvCI (166.7 U/mL, 83.3 U/mL and 0 U/mL, respectively). All the three samples were applied with the same concentrations of the fuel strand L_1, 0.02 μM and the time-dependent fluorescence changes were recorded with a Cary Eclipse Fluorometer (Varian Inc.) at λ_{em} = 665 nm (37 °C).

Dynamic transcription of the DFHBI aptamer from the nickase guided transient transcription machinery

The transient reaction module for the transcription of the DFHBI aptamer was a mixture of the DNA template T_7/A_5 (0.04 μM), T7 RNAP (1.25 × 10^3 U/mL), Nb.BtsI (56.4 U/mL), DFHBI (8 μM), the NTPs (4 mM each) in the RNAP reaction buffer (40 mM Tris-HCl, 1 mM DTT, 2 mM spermidine, 20 mM $MgCl_2$, pH 7.9). Variable concentrations of the fuel strand L_2 (0.1 μM, 0.2 μM and 0.4 μM) were applied to the reaction module and the dynamic time-dependent fluorescence changes of the DFHBI-aptamer complex were recorded with a Cary Eclipse Fluorometer (Varian Inc.) at λ_{em} = 500 nm (37 °C).

For the cyclic transient transcription of the DFHBI aptamer, the reaction module including the DNA template T_7/A_5 (0.04 μM), T7 RNAP (1.25 × 10^3 U/mL), Nb.BtsI (56.4 U/mL), DFHBI (8 μM), and the NTPs (4 mM each) was applied twice with the fuel strand L_2: 0.04 μM and 0.2 μM. The dynamic time-dependent fluorescence changes of the DFHBI-aptamer complex were recorded with a Cary Eclipse Fluorometer (Varian Inc.) at λ_{em} = 500 nm (37 °C).

Transient Gated operations of the nickase guided transient transcription machinery

The mixture of the reaction module contained two DNA templates, T_6/A_4 (0.02 μM) and T_7/A_5 (0.06 μM), and the T7 RNAP (1.25 × 10^3 U/mL), Nt.BbvCI (123.9 U/mL), Nb.BtsI (94 U/mL), MG (4 μM), DFHBI (8 μM) and NTPs (4 mM each) in the RNAP reaction buffer (40 mM Tris-HCl, 1 mM

DTT, 2 mM spermidine, 20 mM $MgCl_2$, pH 7.9). The transient transcription of the templates were triggered by adding L_1 (0.25 μM)/L_2 (0.45 μM). The dynamic transcription of the MG and DFHBI aptamers were followed by the time-dependent fluorescence changes of the MG-aptamer complex at λ_{em} = 665 nm and of the DFHBI-aptamer complex at λ_{em} = 500 nm, recording with a Cary Eclipse Fluorometer (Varian Inc.) at 37 °C.

To gate the respective transcription processes, the reaction module was subjected to the blocker (**5**) (0.4 μM) or (**6**) (1.2 μM), for a time interval of 10 min, and the respective dynamic transcription processes were triggered by L_1 (0.25 μM)/L_2 (0.45 μM), followed by monitoring the time-dependent fluorescence changes of the respective RNA aptamer-ligand complexes using a Cary Eclipse Fluorometer (Varian Inc.) at 37 °C.

Transient Gated transcription of DNAzymes and programmed catalysis

For the dynamic transcription of the DNAzymes, the mixture of the reaction module included two DNA templates, T_8/A_6 (0.02 μM) and T_9/A_7 (0.08 μM), and the T7 RNAP (1.67 × 10^3 U/mL), Nt.BbvCI (166.7 U/mL), Nb.BtsI (133.3 U/mL) and NTPs (4 mM each) in the RNAP reaction buffer (40 mM Tris-HCl, 1 mM DTT, 2 mM spermidine, 20 mM $MgCl_2$, pH 7.9). The transient transcription of the DNAzymes were triggered by adding L_1 (0.2 μM)/L_2 (0.64 μM) at 37 °C. To gate the respective transcription processes, the reaction module was subjected to the blocker (**5**) (0.4 μM) or (**6**) (1.6 μM).

Samples of 100 μL solutions of the transcription system were collected at different time-intervals and applied with the couplers (P_1/Q_1, P_2/Q_2, 1 μM each), the DNAzyme subunits (X, Y, Z and U, 1 μM each) and the substrates (S_1 and S_2, 2 μM each) and the time-dependent fluorescence changes of the respective fluorophore labeled fragmented substrates were followed (FAM, λ_{ex} = 495 nm, λ_{em} = 518 nm; Cy5, λ_{ex} = 635 nm, λ_{em} = 665 nm) at 25 °C.

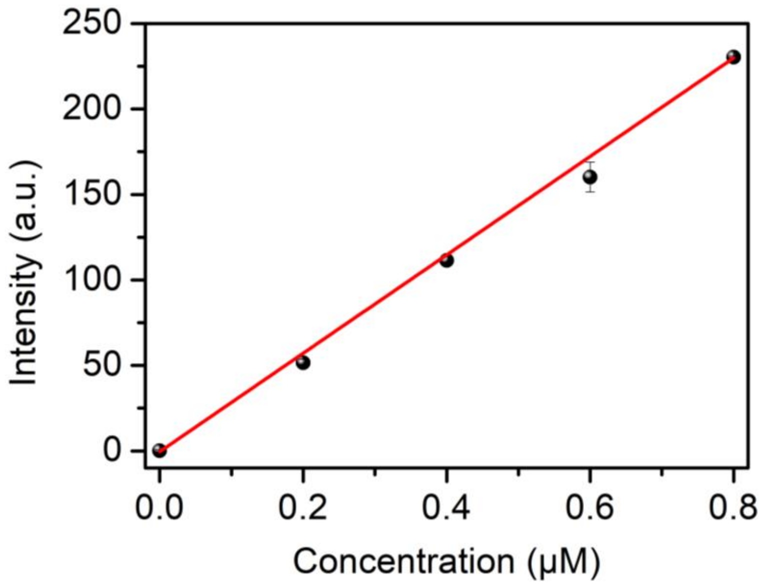

Figure S1. Calibration curve corresponding to the fluorescence intensities of MG-RNA aptamer complex, MG (4 µM), in the presence of variable concentrations of the RNA aptamer. The curve is fit linearly and the $r^2 = 0.9996$.

Kinetic model and computational simulation of the temporal transient transcription of the MG-RNA aptamer by the Pb^{2+}-ion-dependent DNAzyme-triggered transcription machinery.

To further, quantitatively, evaluate the temporal transcription of the MG-RNA aptamer according to Figure 1(A), we formulated a kinetic model that follows the stepwise reaction associated with the triggered activation of the reaction module and the subsequent transcription of the RNA product and the concomitant DNAzyme driven depletion of the reaction intermediates to recover the parent "rest" reaction module. The scheme of the stepwise reactions comprising the model are displayed in Figure S2.

To quantitatively, computationally simulate the experimental temporal fluorescence changes generated by the MG-RNA aptamer complex, the temporal fluorescence changes shown in Figure 1(B) were translated into temporal concentrations of the MG-RNA aptamer using an appropriate calibration curve, Figure S1. Figure S3, curves (i), (ii), and (iii) depict the experimental temporal concentration changes of the MG-RNA aptamer generated by different concentrations of the fuel strand (dotted curve). Curve (i) was then computationally fitted to the kinetic model displayed in Figure S2. The fitted simulated curve is displayed in Figure S3, curve i', (solid curve). The derived rate constants are presented in Table S1. The rate constants were, then, applied to simulate the experimental curves (ii) and (iii) and the simulated temporal curves are displayed in curves (ii') and (iii') (solid curve). The fit of simulated curves to the experimental results using the rate constants derived for curve (i), suggest that the kinetic model and the set of rate constants presented adequately the kinetics of the transient transcription processes.

Kinetic equations of the dissipative DNAzyme-triggered operation of the transient transcription machinery shown in Figure 1:

$$T_1A_1 + L_r \underset{k_{-1}}{\overset{k_1}{\rightleftharpoons}} T_1A_1L_r \quad (1)$$

$$T_1A_1L_r + RNAP \underset{k_{-2}}{\overset{k_2}{\rightleftharpoons}} T_1A_1L_r \bullet RNAP \quad (2)$$

$$T_1A_1L_r \bullet RNAP + NTPs \xrightarrow{k_3} T_1A_1L_r + RNAP + R_1 \quad (3)$$

$$T_1A_1L_r \xrightarrow{k_4} T_1A_1L_{r\text{-}1}L_{r\text{-}2} \quad (4)$$

$$T_1A_1L_{r\text{-}1}L_{r\text{-}2} \underset{k_{-5}}{\overset{k_5}{\rightleftharpoons}} T_1A_1 + L_{r\text{-}1} + L_{r\text{-}2} \quad (5)$$

Derivatives:

$$\frac{dT_1A_1}{dt} = -k_1[T_1A_1][L_r] + k_{-1}[T_1A_1L_r] + k_5[T_1A_1L_{r\text{-}1}L_{r\text{-}2}] - k_{-5}[T_1A_1][L_{r\text{-}1}][L_{r\text{-}2}]$$

$$\frac{dL_r}{dt} = -k_1[T_1A_1][L_r] + k_{-1}[T_1A_1L_r]$$

$$\frac{dT_1A_1L_r}{dt} = k_1[T_1A_1][L_r] - k_{-1}[T_1A_1L_r] - k_2[T_1A_1L_r][RNAP] + k_{-2}[T_1A_1L_r\bullet RNAP] + k_3[T_1A_1L_r\bullet RNAP][NTPs] - k_4[T_1A_1L_r]$$

$$\frac{dRNAP}{dt} = -k_2[T_1A_1L_r][RNAP] + k_{-2}[T_1A_1L_r\bullet RNAP] + k_3[T_1A_1L_r\bullet RNAP][NTPs]$$

$$\frac{dT_1A_1L_r\bullet RNAP}{dt} = k_2[T_1A_1L_r][RNAP] - k_{-2}[T_1A_1L_r\bullet RNAP] - k_3[T_1A_1L_r\bullet RNAP][NTPs]$$

$$\frac{dNTPs}{dt} = -k_3[T_1A_1L_r\bullet RNAP][NTPs]$$

$$\frac{dR_1}{dt} = k_3[T_1A_1L_r\bullet RNAP][NTPs]$$

$$\frac{dT_1A_1L_{r-1}L_{r-2}}{dt} = k_4[T_1A_1L_r] - k_5[T_1A_1L_{r\text{-}1}L_{r\text{-}2}] + k_{-5}[T_1A_1][L_{r\text{-}1}][L_{r\text{-}2}]$$

$$\frac{dL_{r-1}}{dt} = k_5[T_1A_1L_{r\text{-}1}L_{r\text{-}2}] - k_{-5}[T_1A_1][L_{r\text{-}1}][L_{r\text{-}2}]$$

$$\frac{dL_{r-2}}{dt} = k_5[T_1A_1L_{r\text{-}1}L_{r\text{-}2}] - k_{-5}[T_1A_1][L_{r\text{-}1}][L_{r\text{-}2}]$$

Figure S2. Computational simulation of the dissipative DNAzyme-triggered operation of a transient transcription machinery shown in Figure 1. The kinetic scheme of the sub-reactions associated with the time-dependent concentration changes during the dissipative transitions is summarized in the above equations. Knowing the time-dependent concentration changes of the RNA product R_1, we computationally simulated the time-dependent concentration changes by using Matlab R2020a.

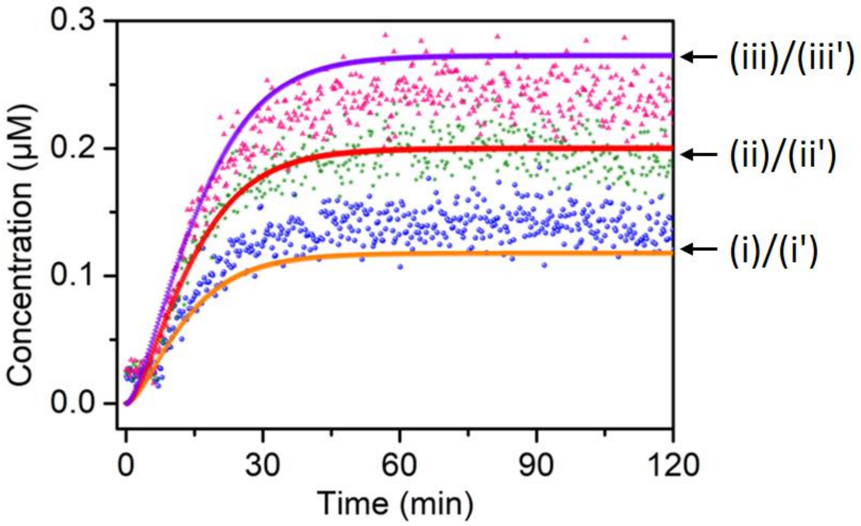

Figure S3. Temporal concentration changes of the MG-RNA aptamer generated by the transient reaction module shown in Figure 1(A) in the presence of variable concentrations of the fuel triggering strand L_r: (i) 0.05 μM; (ii) 0.1 μM; (iii) 0.15 μM. Solid curves (i', ii', and iii') correspond to the computationally simulated kinetic profiles. Dotted curves (i, ii, and iii) represent the experimental data.

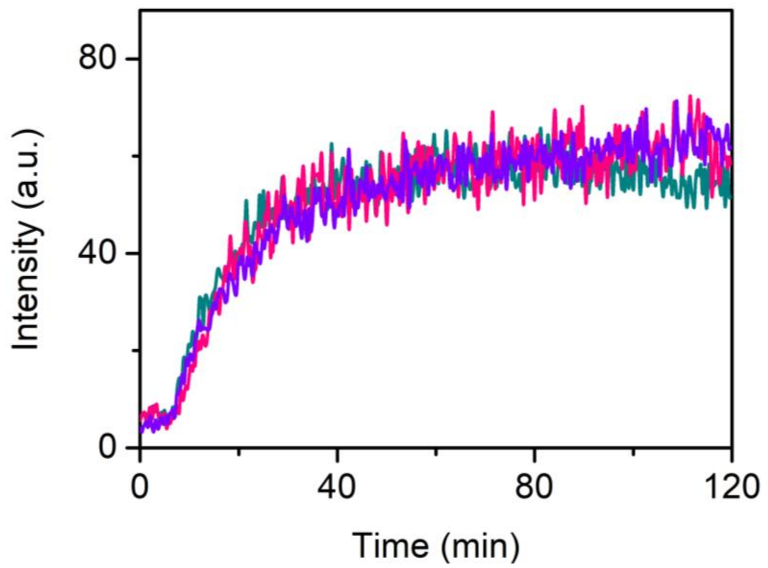

Figure S4. Temporal fluorescence intensities of the MG-RNA aptamer generated by the DNAzyme-triggered transient reaction module shown in Figure 1(A), with the fuel triggering strand Lr 0.1 μM, experiments repeated for 3 times.

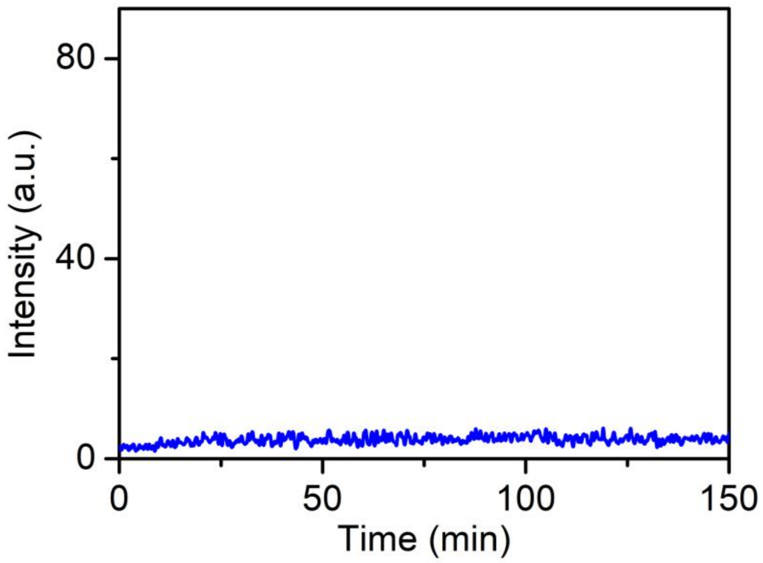

Figure S5. Control experiment for the temporal fluorescence intensities of the MG-RNA aptamer generated by the DNAzyme-triggered transient reaction module shown in Figure 1(A) in the absence of the trigger L_r.

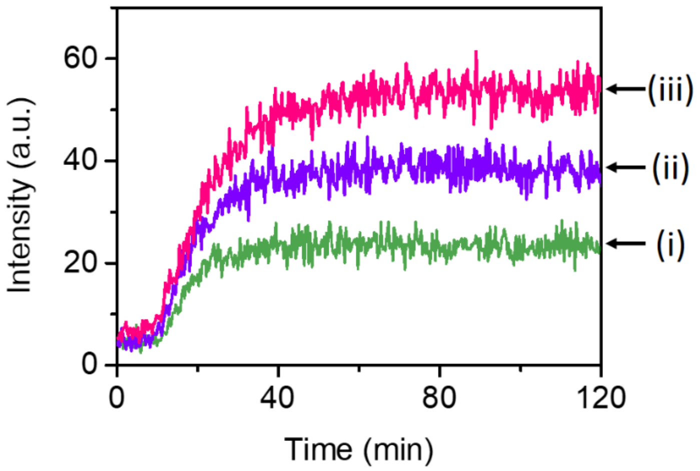

Figure S6. Temporal fluorescence intensities of the MG-RNA aptamer generated by the transient reaction module shown in Figure 1(A) in the presence of variable concentrations of different concentrations of NTPs: (i) 2 mM; (ii) 4 mM; (iii) 6 mM.

A control experiment employing non ribonucleobase containing promoter-triggering strand, L_c, to activate the reaction module transcribing the MG-RNA aptamer was investigated, Figure S7(A). Figure S7(B) curve (i) depicts the time-dependent fluorescence changes proceeding in the system. A non-dissipative, continuous generation of the MG-RNA aptamer is observed. For comparison, Figure S7(B), curve (ii) depicts the temporal fluorescence changes of the MG-RNA aptamer in the transiently-modulated system shown in Figure 1(A). The results indicate that the ribonucleobase in the triggering strand L_r is essential to drive the Pb^{2+}-DNAzyme-dependent transiently-modulated transcription of the MG-RNA aptamer.

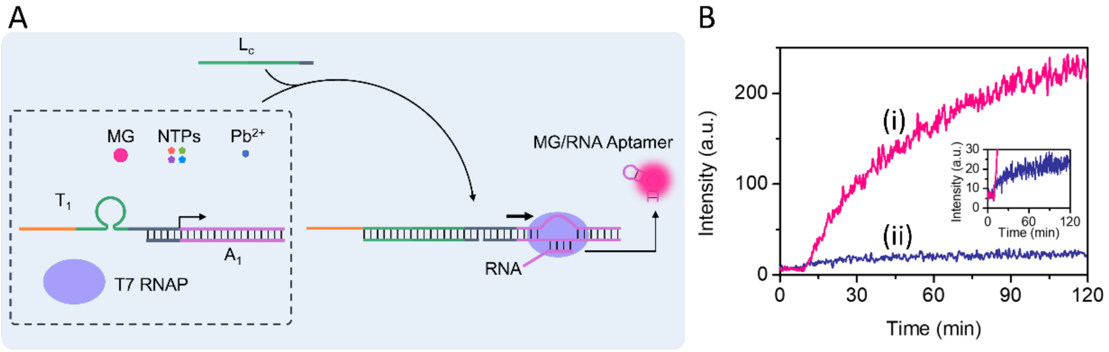

Figure S7. (A) Schematic reaction module for the DNAzyme-triggered operation of a transcription machinery synthesizing the MG-RNA aptamer by using the control fuel strand L_c demonstrating that the dissipative dynamic transcription of the MG-RNA aptamer is dependent on the DNAzyme cleavage of the trigger. (B) Time-dependent fluorescent changes of the MG-RNA aptamer complexes from the transcription machinery by using the different trigger L_c (0.1 µM, curve (i)) and L_r (0.1 µM, curve (ii)), respectively. Inset is the magnified curve (ii).

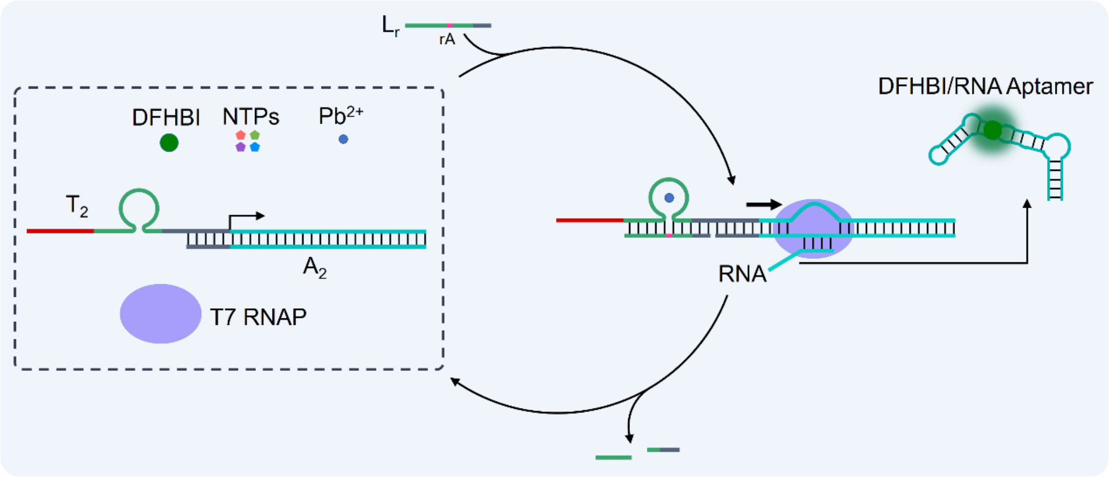

Figure S8. Schematic reaction module for the DNAzyme-triggered operation of a transient transcription machinery synthesizing the DFHBI-RNA aptamer.

Kinetic model and computational simulation of the temporal transient transcription of the MG-RNA aptamer by the strand-displacement-stimulated transcription machinery.

To further, quantitatively, evaluate the temporal transcription of the MG-RNA aptamer according to Figure 3(A), we formulated a kinetic model that follows the stepwise reaction associated with the triggered activation of the reaction module and the subsequent transcription of the RNA product and the concomitant strand-displacement driven depletion of the reaction intermediates to recover the parent “rest” reaction module. The scheme of the stepwise reactions comprising the model are displayed in Figure S9.

To quantitatively, computationally simulated the experimental temporal fluorescence changes generated by the MG-RNA aptamer complex, the temporal fluorescence changes shown in Figure 3(B) were translated into temporal concentrations of the MG-RNA aptamer using an appropriate calibration curve, Figure S1. Figure S10, curves (i), (ii), and (iii) depict the experimental temporal concentration changes of the MG-RNA aptamer generated by different concentrations of the fuel strand (dotted curve). Curve (i) was then computationally fitted to the kinetic model displayed in Figure S9. The fitted simulated curve is displayed in Figure S10, curve i', (solid curve). The derived rate constants are presented in Table S2. The rate constants were, then, applied to simulate the experimental curves (ii) and (iii) and the simulated temporal curves are displayed in curves (ii') and (iii') (solid curve). The fit of simulated curves to the experimental results using the rate constants derived for curve (i), suggest that the kinetic model and the set of rate constants presented adequately the kinetics of the transient transcription processes.

Kinetic equations of the strand-displacement guided the transient transcription machinery shown in Figure 3:

$$\mathrm{T_3A_1 + F \underset{k_{-6}}{\overset{k_6}{\rightleftharpoons}} T_3A_1F} \quad (6)$$

$$\mathrm{T_4A_3 + F \underset{k_{-7}}{\overset{k_7}{\rightleftharpoons}} T_4A_3F} \quad (7)$$

$$\mathrm{T_3A_1F + RNAP \underset{k_{-8}}{\overset{k_8}{\rightleftharpoons}} T_3A_1F \bullet RNAP} \quad (8)$$

$$\mathrm{T_4A_3F + RNAP \underset{k_{-8}}{\overset{k_8}{\rightleftharpoons}} T_4A_3F \bullet RNAP} \quad (9)$$

$$\mathrm{T_3A_1F \bullet RNAP + NTPs \xrightarrow{k_9} T_3A_1F + RNAP + R_2} \quad (10)$$

$$\mathrm{T_4A_3F \bullet RNAP + NTPs \xrightarrow{k_{10}} T_4A_3F + RNAP + F'} \quad (11)$$

$$\mathrm{T_3A_1F + F' \underset{k_{-11}}{\overset{k_{11}}{\rightleftharpoons}} FF' + T_3A_1} \quad (12)$$

$$\mathrm{T_4A_3F + F' \underset{k_{-12}}{\overset{k_{12}}{\rightleftharpoons}} FF' + T_4A_3} \quad (13)$$

Derivatives:

$$\frac{dT_3A_1}{dt} = -k_6[\mathrm{T_3A_1}][\mathrm{F}] + k_{-6}[\mathrm{T_3A_1F}] + k_{11}[\mathrm{T_3A_1F}][\mathrm{F'}] - k_{-11}[\mathrm{FF'}][\mathrm{T_3A_1}]$$

$$\frac{dF}{dt} = -k_6[\mathrm{T_3A_1}][\mathrm{F}] + k_{-6}[\mathrm{T_3A_1F}] - k_7[\mathrm{T_4A_3}][\mathrm{F}] + k_{-7}[\mathrm{T_4A_3F}]$$

$$\frac{dT_3A_1F}{dt} = k_6[\mathrm{T_3A_1}][\mathrm{F}] - k_{-6}[\mathrm{T_3A_1F}] - k_8[\mathrm{T_3A_1F}][\mathrm{RNAP}] + k_{-8}[\mathrm{T_3A_1F{\bullet}RNAP}] + k_9[\mathrm{T_3A_1F{\bullet}RNAP}][\mathrm{NTPs}] - k_{11}[\mathrm{T_3A_1F}][\mathrm{F'}] + k_{-11}[\mathrm{FF'}][\mathrm{T_3A_1}]$$

$$\frac{dT_4A_3}{dt} = -k_7[\mathrm{T_4A_3}][\mathrm{F}] + k_{-7}[\mathrm{T_4A_3F}] + k_{12}[\mathrm{T_4A_3F}][\mathrm{F'}] - k_{-12}[\mathrm{FF'}][\mathrm{T_4A_3}]$$

$$\frac{dT_4A_3F}{dt} = k_7[\mathrm{T_4A_3}][\mathrm{F}] - k_{-7}[\mathrm{T_4A_3F}] - k_8[\mathrm{T_4A_3F}][\mathrm{RNAP}] + k_{-8}[\mathrm{T_4A_3F{\bullet}RNAP}] + k_{10}[\mathrm{T_4A_3F{\bullet}RNAP}][\mathrm{NTPs}] - k_{12}[\mathrm{T_4A_3F}][\mathrm{F'}] + k_{-12}[\mathrm{FF'}][\mathrm{T_4A_3}]$$

$$\frac{dRNAP}{dt} = -k_8[\mathrm{T_3A_1F}][\mathrm{RNAP}] + k_{-8}[\mathrm{T_3A_1F{\bullet}RNAP}] - k_8[\mathrm{T_4A_3F}][\mathrm{RNAP}] + k_{-8}[\mathrm{T_4A_3F{\bullet}RNAP}] + k_9[\mathrm{T_3A_1F{\bullet}RNAP}][\mathrm{NTPs}] + k_{10}[\mathrm{T_4A_3F{\bullet}RNAP}][\mathrm{NTPs}]$$

$$\frac{dT_3A_1F{\bullet}RNAP}{dt} = k_8[\mathrm{T_3A_1F}][\mathrm{RNAP}] - k_{-8}[\mathrm{T_3A_1F{\bullet}RNAP}] - k_9[\mathrm{T_3A_1F{\bullet}RNAP}][\mathrm{NTPs}]$$

$$\frac{dT_4A_3F{\bullet}RNAP}{dt} = k_8[\mathrm{T_4A_3F}][\mathrm{RNAP}] - k_{-8}[\mathrm{T_4A_3F{\bullet}RNAP}] - k_{10}[\mathrm{T_4A_3F{\bullet}RNAP}][\mathrm{NTPs}]$$

$$\frac{dNTPs}{dt} = -k_9[\mathrm{T_3A_1F{\bullet}RNAP}][\mathrm{NTPs}] - k_{10}[\mathrm{T_4A_3F{\bullet}RNAP}][\mathrm{NTPs}]$$

$$\frac{dR_2}{dt} = k_9[\mathrm{T_3A_1F{\bullet}RNAP}][\mathrm{NTPs}]$$

$$\frac{dF'}{dt} = k_{10}[\mathrm{T_4A_3F{\bullet}RNAP}][\mathrm{NTPs}] - k_{11}[\mathrm{T_3A_1F}][\mathrm{F'}] + k_{-11}[\mathrm{FF'}][\mathrm{T_3A_1}] - k_{12}[\mathrm{T_4A_3F}][\mathrm{F'}] + k_{-12}[\mathrm{FF'}][\mathrm{T_4A_3}]$$

$$\frac{dFF'}{dt} = k_{11}[\mathrm{T_3A_1F}][\mathrm{F'}] - k_{-11}[\mathrm{FF'}][\mathrm{T_3A_1}] + k_{12}[\mathrm{T_4A_3F}][\mathrm{F'}] - k_{-12}[\mathrm{FF'}][\mathrm{T_4A_3}]$$

Figure S9. Computational simulation of the strand-displacement guided transient transcription machinery shown in Figure 3. The kinetic scheme of the sub-reactions associated with the time-dependent concentration changes during the dissipative transitions is summarized in the above equations. Knowing the time-dependent concentration changes of the RNA product R_2, we computationally simulated the time-dependent concentration changes by using Matlab R2020a.

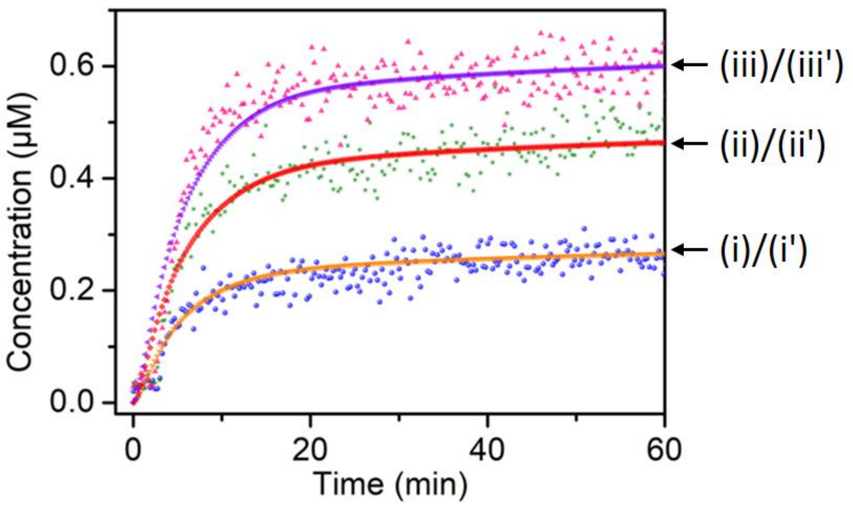

Figure S10. Temporal concentration changes corresponding to the transcribed MG-RNA aptamer following scheme shown in Figure 3(A) in the presence of variable concentrations of fuel F: (i) 0.05 μM; (ii) 0.1 μM; (iii) 0.15 μM. Solid curves (i', ii', and iii') correspond to the computationally simulated kinetic profiles. Dotted curves (i, ii, and iii) represent the experimental data.

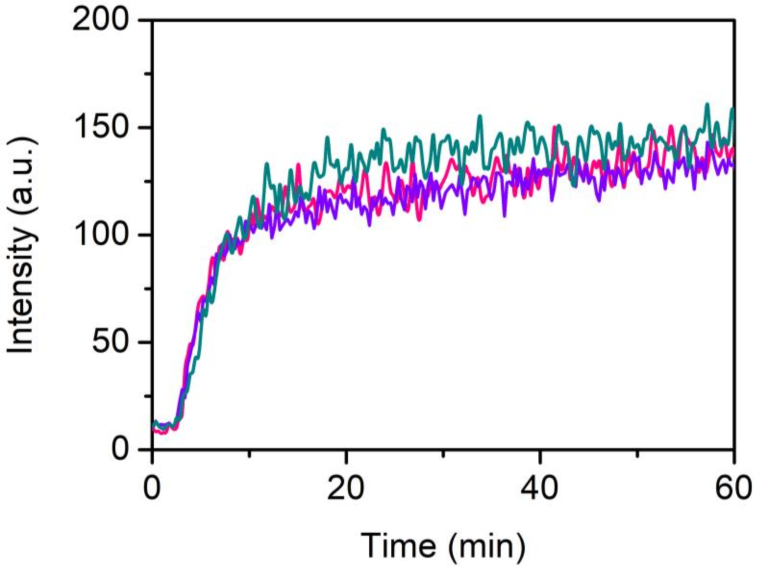

Figure S11. Temporal fluorescence intensities of the MG-RNA aptamer generated by the strand-displacement guided transient reaction module shown in Figure 3(A), in the presence of trigger F 0.1 μM, experiments repeated for 3 times.

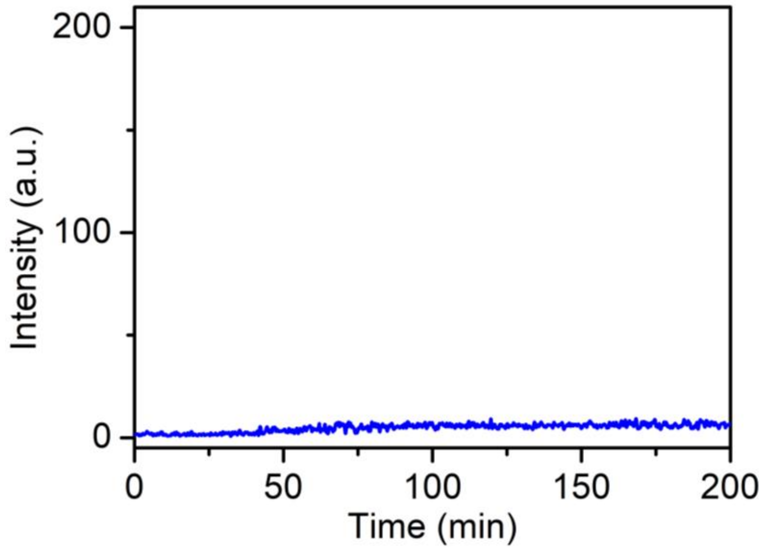

Figure S12. Control experiment for the temporal fluorescence intensities of the MG-RNA aptamer generated by the strand-displacement guided transient reaction module shown in Figure 3(A) in the absence of the trigger F.

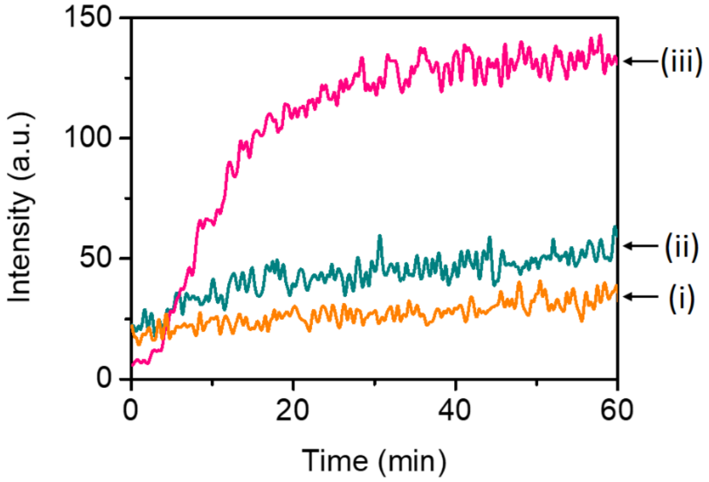

Figure S13. Temporal fluorescence changes corresponding to the transcribed MG-RNA aptamer shown in Figure 3(A) using the strand displacement principle in the presence of variable concentrations of NTPs: (i) 1 mM; (ii) 2 mM; (iii) 4 mM.

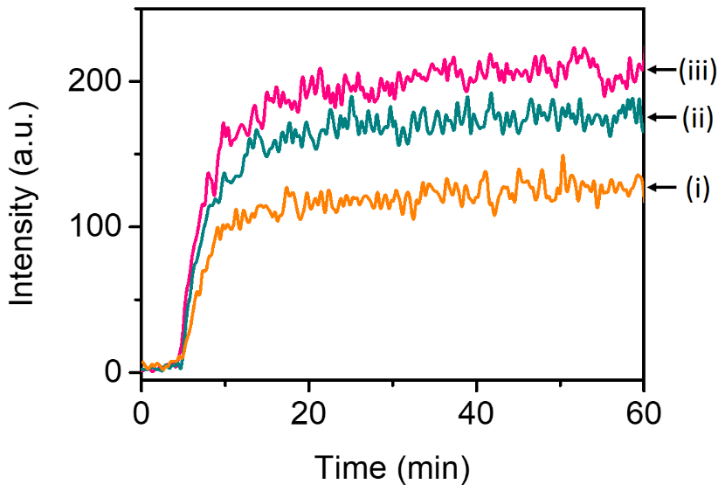

Figure S14. Temporal fluorescence changes corresponding to the transcribed MG-RNA aptamer shown in Figure 3(A) using the strand displacement principle in the presence of variable concentrations of the DNA template T_4/A_3: (i) 0.1 μM; (ii) 0.05 μM; (iii) 0.02 μM.

PAGE electrophoretic characterization of the fueled transient operation of the transcription machinery T_4/A_3 shown in Figure 3(A).

Due to the presence of the two transcription machineries T_3/A_1 and T_4/A_3 and the F-fueled activation of the two transcription machineries, the clear separation of the temporally formed concomitantly operating machineries was difficult, and hence only the transient operation of the transcription template T_4/A_3 was executed.

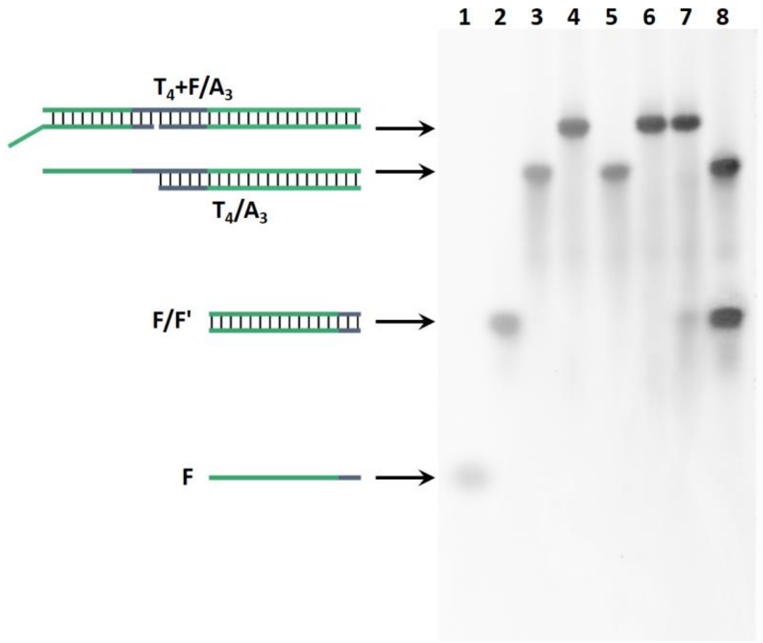

Figure S15. PAGE electrophoretic separation demonstrating the fueled transient operation of the T_4/A_3 transcription template transcribing the anti-fuel strand F' and the temporal formation of the F/F' waste product: (Lane 1-Lane 4: reference constituents of the transcription machinery) Lane 1-the fuel strand F (2 µM, 10 µL); Lane 2-the reference waste product (1 µM, 10 µL); Lane 3-the template T_4/A_3 (1 µM, 10 µL), Lane 4-the F-modified T_4/A_3 (1 µM, 10 µL); (Lane 5-Lane 8: dynamic, temporal features of the transcription machinery constituents: T_4/A_3, 1 µM; NTPs, 4 mM each) Lane 5-the transcription template T_4/A_3 prior to the addition of the fuel strand F and T7 RNAP, in the presence of NTPs; Lane 6-After addition of the fuel strand F (1 µM), in the presence of NTPs; Lane 7-the system upon addition of T7 RNAP (1.25×10^4 U/mL), in the presence of NTPs, after a time-interval of two minutes; Lane 8-the system in the presence of T7 RNAP, the NTPs, after a time interval of one hour. The formation of the waste product F/F' and the recovery of the inactive T_4/A_3 template confirm the transient operation of the fueled transcription machinery generating F/F' as waste. The 8% PAGE was performed for 20 h at 80 V (at 8 ℃).

To support the strand displacement process as a guiding principle to modulate the transcription machinery, we engineered a reaction module, Figure S16(A), Where the promoter-triggering strand Fc, forms a stable transcription template T_3/Fc+A_1, where Fc **cannot** be displaced by the transcribed product F' that prohibiting the transiently-modulated transcription machinery of T_3/A_1. Figure S16(B), curve (i) shows the temporal fluorescence changes of the MG-RNA aptamer upon operation of the transcription machinery displayed in Figure S16(A). Continuous, non-dissipative formation of the MG-RNA aptamer is observed. Figure S16(B), curve (ii) shows the temporal fluorescence changes of the MG-RNA aptamer transcribed according to Figure 3(A). The results demonstrate that the strand displacement of the triggering strand F by the strand F' is essential to induce the transiently-modulated transcription apparatus shown in Figure 3(A).

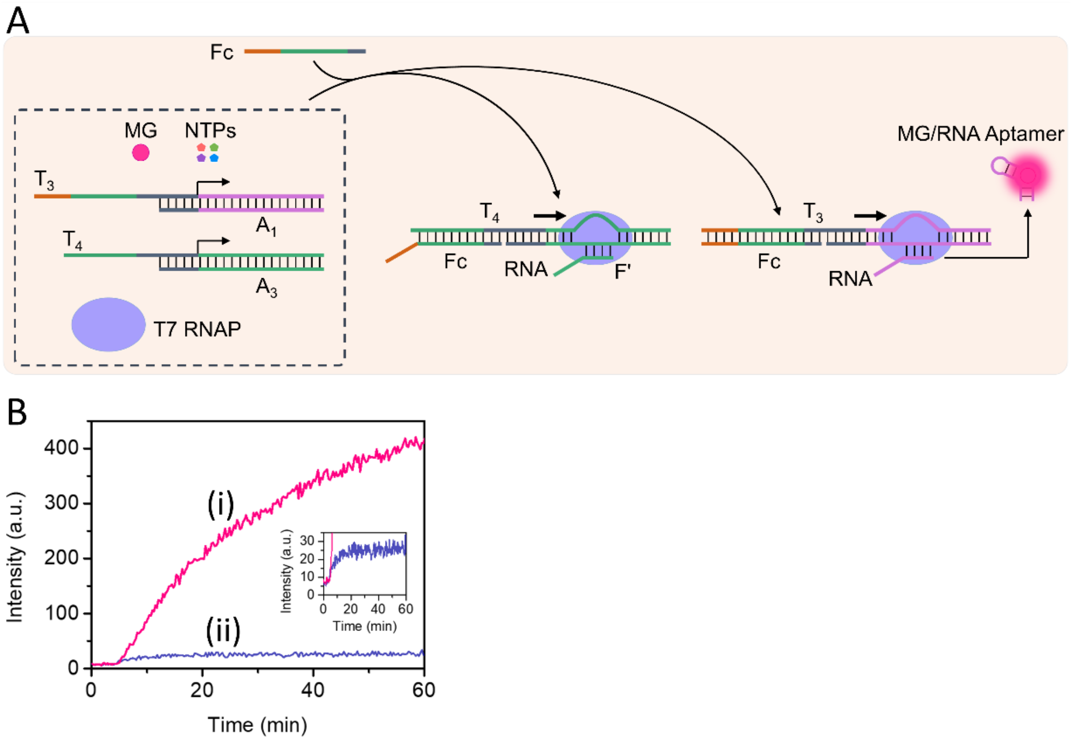

Figure S16. (A) Schematic of the dynamic transcription of the MG-RNA aptamer based on the strand-displacement guided transient transcription machinery by using the control trigger Fc that cannot be displaced by the transcribed F'. (B) Time-dependent fluorescent changes of the MG-RNA aptamer complexes based on the strand displacement principle by using the different triggers: Fc (0.1 μM, curve (i)) and F (0.1 μM, curve (ii)), respectively. Inset is the magnified curve (ii).

Furthermore, as the transient template T_4/A_3 includes complementary domain to the fuel strand F, it is important to prove that the template T_4/A_3 stay intact in the presence of F. This is confirmed by the gel electrophoretic experiment presented in Figure S17. This experiment demonstrates that the addition of F to the active template T_4+F/A_3 does not yield any displaced product.

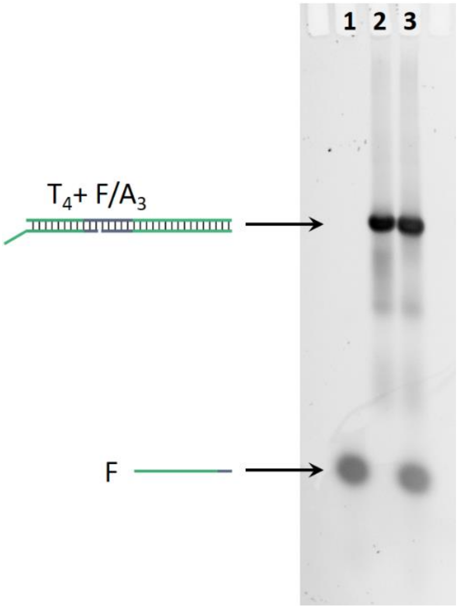

Figure S17. The PAGE gel image of the fuel strand F and the T_4/A_3 DNA template demonstrating that the fuel F only binds to the promoter segment and cannot displace the coding segment of the T_4/A_3 DNA template: Lane **1**: F (2 µM, 10 µL); Lane **2**: T_4+F/A_3 (0.5 µM, 10 µL); Lane **3**: T_4+F/A_3 (0.5 µM) + F (2 µM), 10 µL. The 10% PAGE was performed for 16 h at 80 V (at 8 °C).

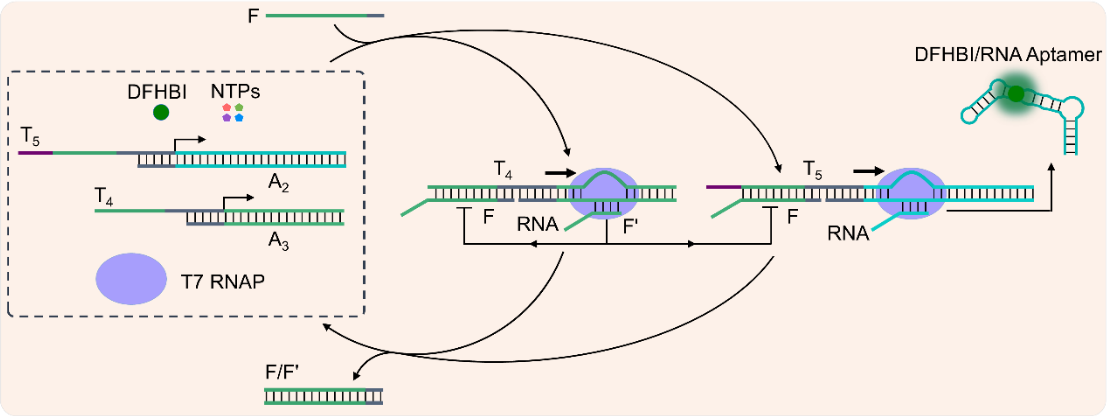

Figure S18. Schematic of the dynamic transcription of the DFHBI-RNA aptamer using the strand displacement principle guided transient transcription machinery.

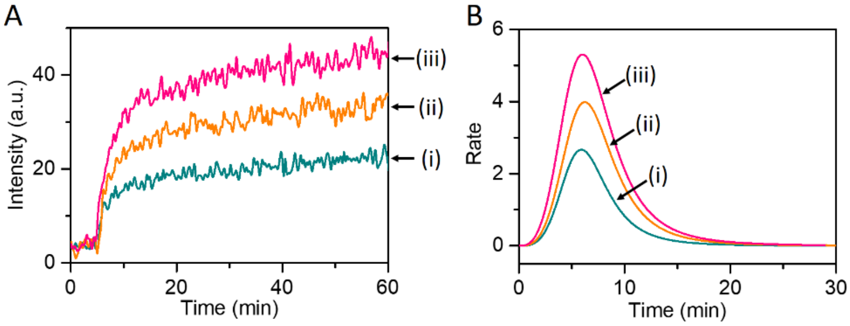

Figure S19. (A) Temporal fluorescence changes corresponding to the transcribed DFHBI-RNA aptamer using the strand displacement principle shown in Figure S18 in the presence of variable concentrations of fuel F: (i) 0.05 μM; (ii) 0.1 μM; (iii) 0.15 μM. (B) Time-dependent catalytic transcription rates corresponding to the transient synthesis of the DFHBI-RNA aptamer in the presence of different concentrations of fuel F: (i) 0.05 μM; (ii) 0.1 μM; (iii) 0.15 μM.

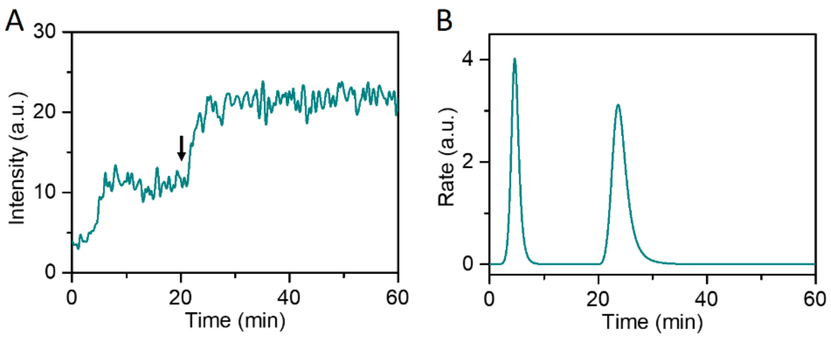

Figure S20. (A) Temporal fluorescence changes upon the cyclic operation of the transient reaction module synthesizing the DFHBI-RNA aptamer. The time marked with an arrow indicates the time reactivation of the reaction module by adding the fuel strand F: 0.05 μM and 0.15 μM. (B) Cyclic catalytic rates corresponding to the stepwise operation of the transient transcription machinery.

Kinetic model and computational simulation of the temporal transient transcription of the MG-RNA aptamer by the nickase-driven transcription machinery.

To further, quantitatively, evaluate the temporal transcription of the MG-RNA aptamer according to Figure 5(A), we formulated a kinetic model that follows the stepwise reaction associated with the triggered activation of the reaction module and the subsequent transcription of the RNA product and the concomitant nickase-driven depletion of the reaction intermediates to recover the parent “rest” reaction module. The scheme of the stepwise reactions comprising the model are displayed in Figure S21.

To quantitatively, computationally simulated the experimental temporal fluorescence changes generated by the MG-RNA aptamer complex, the temporal fluorescence changes shown in Figure 5(B) were translated into temporal concentrations of the MG-RNA aptamer using an appropriate calibration curve, Figure S1. Figure S22, curves (i), (ii), and (iii) depict the experimental temporal concentration changes of the MG-RNA aptamer generated by different concentrations of the fuel strand (dotted curve). Curve (i) was then computationally fitted to the kinetic model displayed in Figure S21. The fitted simulated curve is displayed in Figure S22, curve i', (solid curve). The derived rate constants are presented in Table S3. The rate constants were, then, applied to simulate the experimental curves (ii) and (iii) and the simulated temporal curves are displayed in curves (ii') and (iii') (solid curve). The fit of simulated curves to the experimental results using the rate constants derived for curve (i), suggest that the kinetic model and the set of rate constants presented adequately the kinetics of the transient transcription processes.

Kinetic equations of the nickase-stimulated transient operation of the transcription machinery shown in Figure 5:

$$T_6A_4 + L_1 \underset{k_{-13}}{\overset{k_{13}}{\rightleftharpoons}} T_6A_4L_1 \quad (14)$$

$$T_6A_4L_1 + RNAP \underset{k_{-14}}{\overset{k_{14}}{\rightleftharpoons}} T_6A_4L_1 \bullet RNAP \quad (15)$$

$$T_6A_4L_1 \bullet RNAP + NTPs \xrightarrow{k_{15}} T_6A_4L_1 + RNAP + R_3 \quad (16)$$

$$T_6A_4L_1 + E \underset{k_{-16}}{\overset{k_{16}}{\rightleftharpoons}} T_6A_4L_1 \bullet E \quad (17)$$

$$T_6A_4L_1 \bullet E \xrightarrow{k_{17}} T_6A_4L_{1\text{-}1}L_{1\text{-}2} + E \quad (18)$$

$$T_6A_4L_{1\text{-}1}L_{1\text{-}2} \underset{k_{-18}}{\overset{k_{18}}{\rightleftharpoons}} T_6A_4 + L_{1\text{-}1} + L_{1\text{-}2} \quad (19)$$

Derivatives:

$$\frac{dT_6A_4}{dt} = -k_{13}[T_6A_4][L_1] + k_{-13}[T_6A_4L_1] + k_{18}[T_6A_4L_{1\text{-}1}L_{1\text{-}2}] - k_{-18}[T_6A_4][L_{1\text{-}1}][L_{1\text{-}2}]$$

$$\frac{dL_1}{dt} = -k_{13}[T_6A_4][L_1] + k_{-13}[T_6A_4L_1]$$

$$\frac{dT_6A_4L_1}{dt} = k_{13}[T_6A_4][L_1] - k_{-13}[T_6A_4L_1] - k_{14}[T_6A_4L_1][RNAP] + k_{-14}[T_6A_4L_1\bullet RNAP] + k_{15}[T_6A_4L_1\bullet RNAP][NTPs] - k_{16}[T_6A_4L_1][E] + k_{-16}[T_6A_4L_1\bullet E]$$

$$\frac{dRNAP}{dt} = -k_{14}[T_6A_4L_1][RNAP] + k_{-14}[T_6A_4L_1\bullet RNAP] + k_{15}[T_6A_4L_1\bullet RNAP][NTPs]$$

$$\frac{dT_6A_4L_1\bullet RNAP}{dt} = k_{14}[T_6A_4L_1][RNAP] - k_{-14}[T_6A_4L_1\bullet RNAP] - k_{15}[T_6A_4L_1\bullet RNAP][NTPs]$$

$$\frac{dNTPs}{dt} = -k_{15}[T_6A_4L_1\bullet RNAP][NTPs]$$

$$\frac{dR_3}{dt} = k_{15}[T_6A_4L_1\bullet RNAP][NTPs]$$

$$\frac{dE}{dt} = -k_{16}[T_6A_4L_1][E] + k_{-16}[T_6A_4L_1\bullet E] + k_{17}[T_6A_4L_1\bullet E]$$

$$\frac{dT_6A_4L_1\bullet E}{dt} = k_{16}[T_6A_4L_1][E] - k_{-16}[T_6A_4L_1\bullet E] - k_{17}[T_6A_4L_1\bullet E]$$

$$\frac{dT_6A_4L_{1-1}L_{1-2}}{dt} = k_{17}[T_6A_4L_1\bullet E] - k_{18}[T_6A_4L_{1\text{-}1}L_{1\text{-}2}] + k_{-18}[T_6A_4][L_{1\text{-}1}][L_{1\text{-}2}]$$

$$\frac{dL_{1-1}}{dt} = k_{18}[T_6A_4L_{1\text{-}1}L_{1\text{-}2}] - k_{-18}[T_6A_4][L_{1\text{-}1}][L_{1\text{-}2}]$$

$$\frac{dL_{1-2}}{dt} = k_{18}[T_6A_4L_{1\text{-}1}L_{1\text{-}2}] - k_{-18}[T_6A_4][L_{1\text{-}1}][L_{1\text{-}2}]$$

Figure S21. Computational simulation of the nickase-stimulated transient operation of a transcription machinery shown in Figure 5. The kinetic scheme of the sub-reactions associated with the time-dependent concentration changes during the dissipative transitions is summarized in the above equations. Knowing the time-dependent concentration changes of the RNA product R_3, we computationally simulated the time-dependent concentration changes by using Matlab R2020a.

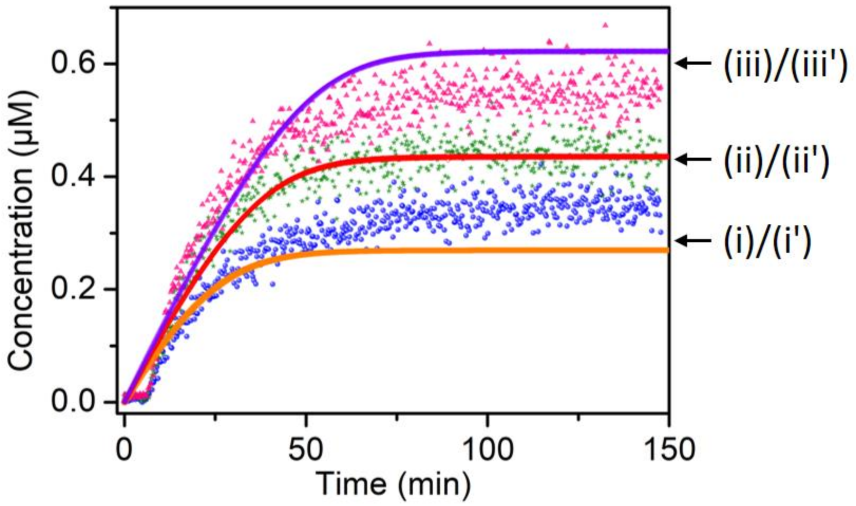

Figure S22. Temporal concentration changes corresponding to the transcription of the MG-RNA aptamer upon the triggered nickase-stimulated transcription machinery, in the presence of variable concentrations of the trigger L_1: (i) 0.1 μM; (ii) 0.2 μM; (iii) 0.3 μM. Solid curves (i', ii', and iii') correspond to the computationally simulated kinetic profiles. Dotted curves (i, ii, and iii) represent the experimental data.

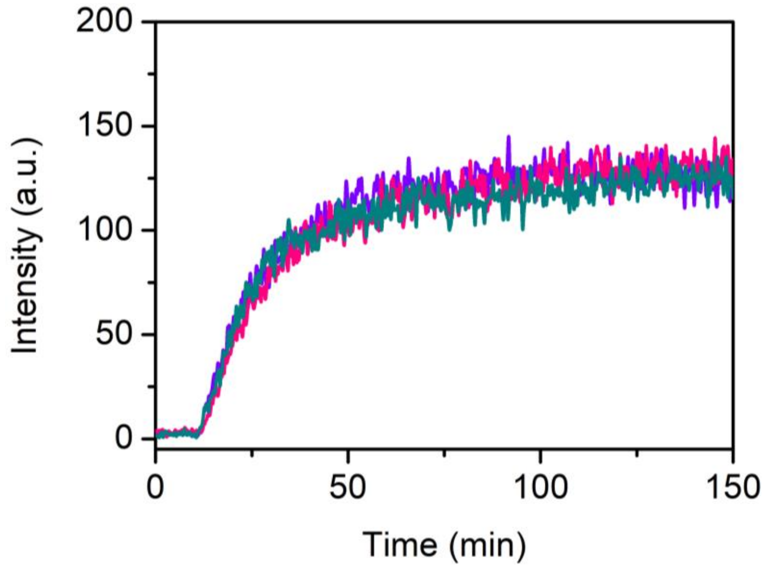

Figure S23. Temporal fluorescence intensities of the MG-RNA aptamer generated by the nickase-stimulated guided transient reaction module shown in Figure 5(A), in the presence of trigger L_1 0.2 μM, experiments repeated for 3 times.

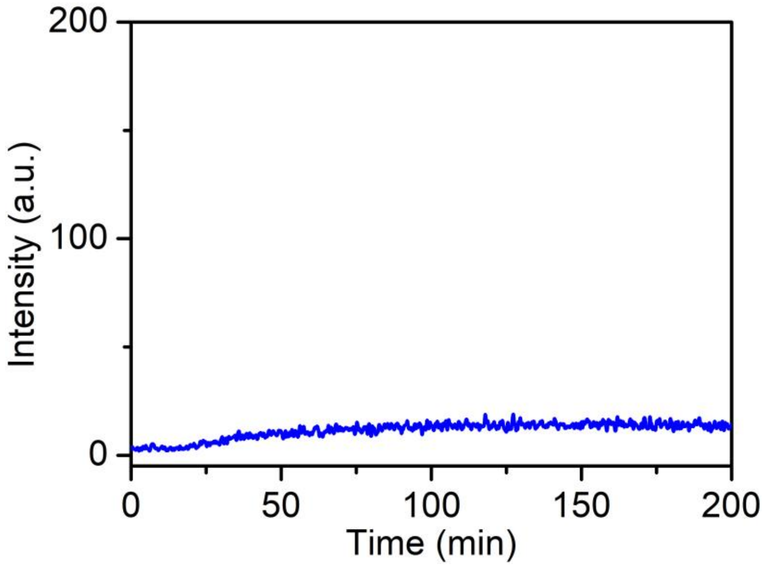

Figure S24. Control experiment for the temporal fluorescence intensities of the MG-RNA aptamer generated by the nickase-stimulated transient reaction module shown in Figure 5(A) in the absence of the trigger L_1.

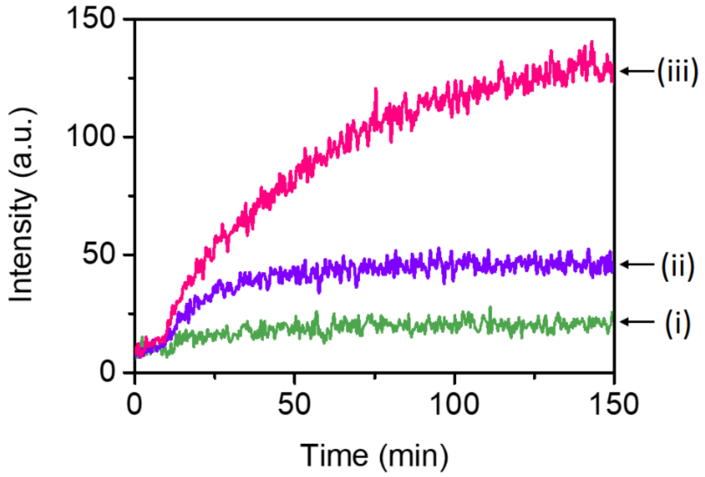

Figure S25. Time-dependent fluorescence changes of the MG-aptamer complex based on the nickase-stimulated dissipative transcription machinery in the presence of different concentrations of NTPs: (i) 1.33 mM; (ii) 2.66 mM; (iii) 4 mM.

The reaction module shown in Figure 5(A) driving the nickase-stimulated transient modulation of the transcription of the MG RNA aptamer was examined in the presence of different concentrations of nickase, Figure S26, curves (i) and (ii). As the concentration of nickase is lower, the temporal transcription efficiency increases. However, in the absence of added nickase, continuous transcription of the MG RNA aptamer is observed, Figure S26, curve (iii), implying that the transcription machinery is not depleted (the slow decrease in the transcription efficiency is due to the depletion of the NTPs). These results emphasizing the significant function of nickase in depletion the active transcription template and the induction of a dissipative transcription process.

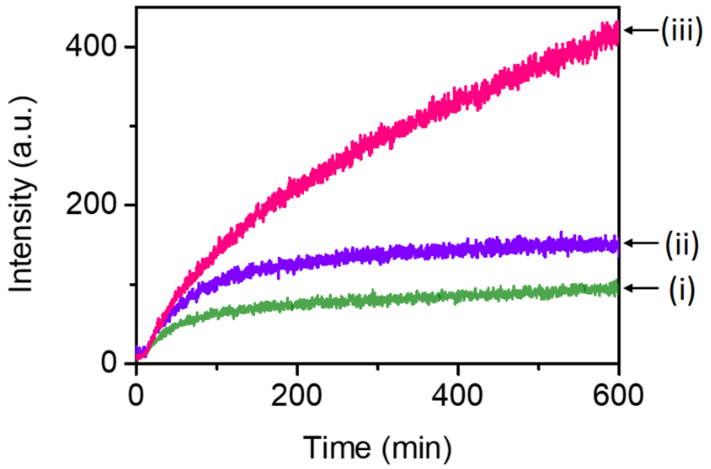

Figure S26. Time-dependent fluorescence changes of the MG-aptamer complex based on the nickase-stimulated dissipative transcription machinery in the presence of different concentrations of Nt.BbvCI: (i) 166.7 U/mL; (ii) 83.3 U/mL; (iii) 0 U/mL.

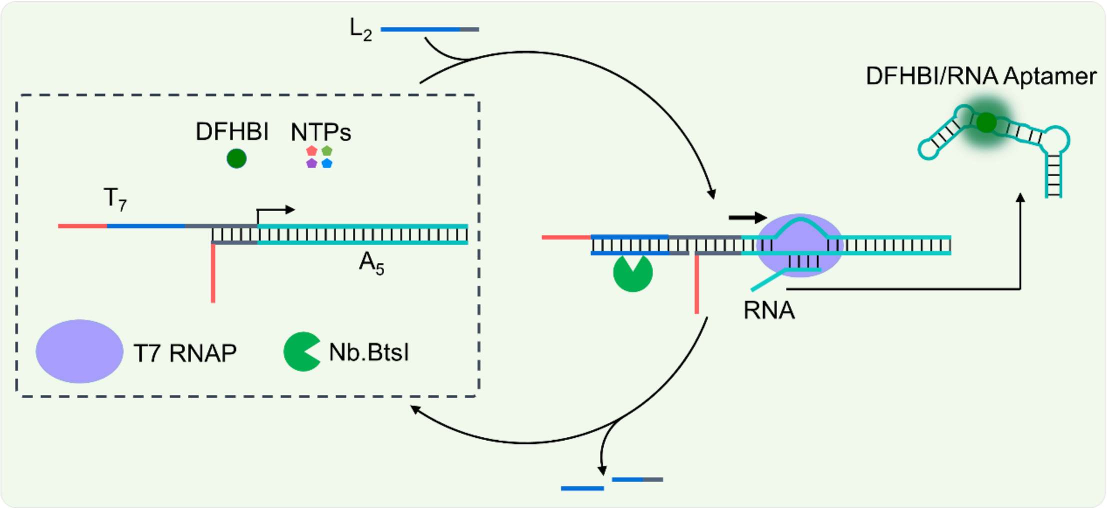

Figure S27. Schematic of the transient transcription of the DFHBI RNA aptamer based on the nickase (Nb.BtsI)-stimulated dissipative transcription machinery.

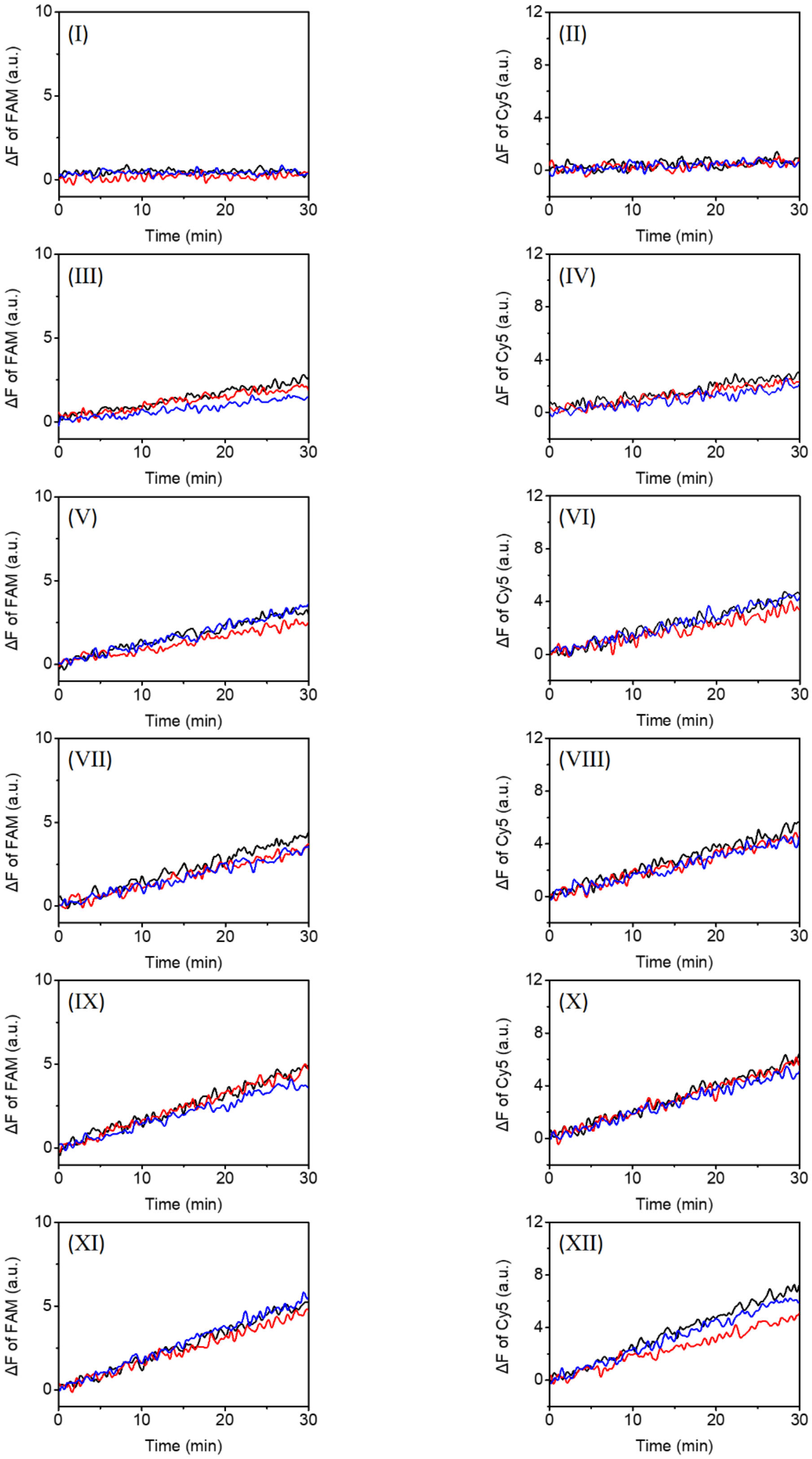

Figure S28. Time-dependent catalytic activities of DNAzyme (**1**) and DNAzyme (**2**) assembled by the transiently-modulated operation of the transcription machineries shown in Figure 7 at different time-intervals: (I-II), 0 min; (III-IV), 10 min; (V-VI), 20 min; (VII-VIII), 30 min; (IX-X), 60 min; (XI-XII), 120 min. Each measurement was repeated three times (N = 3).

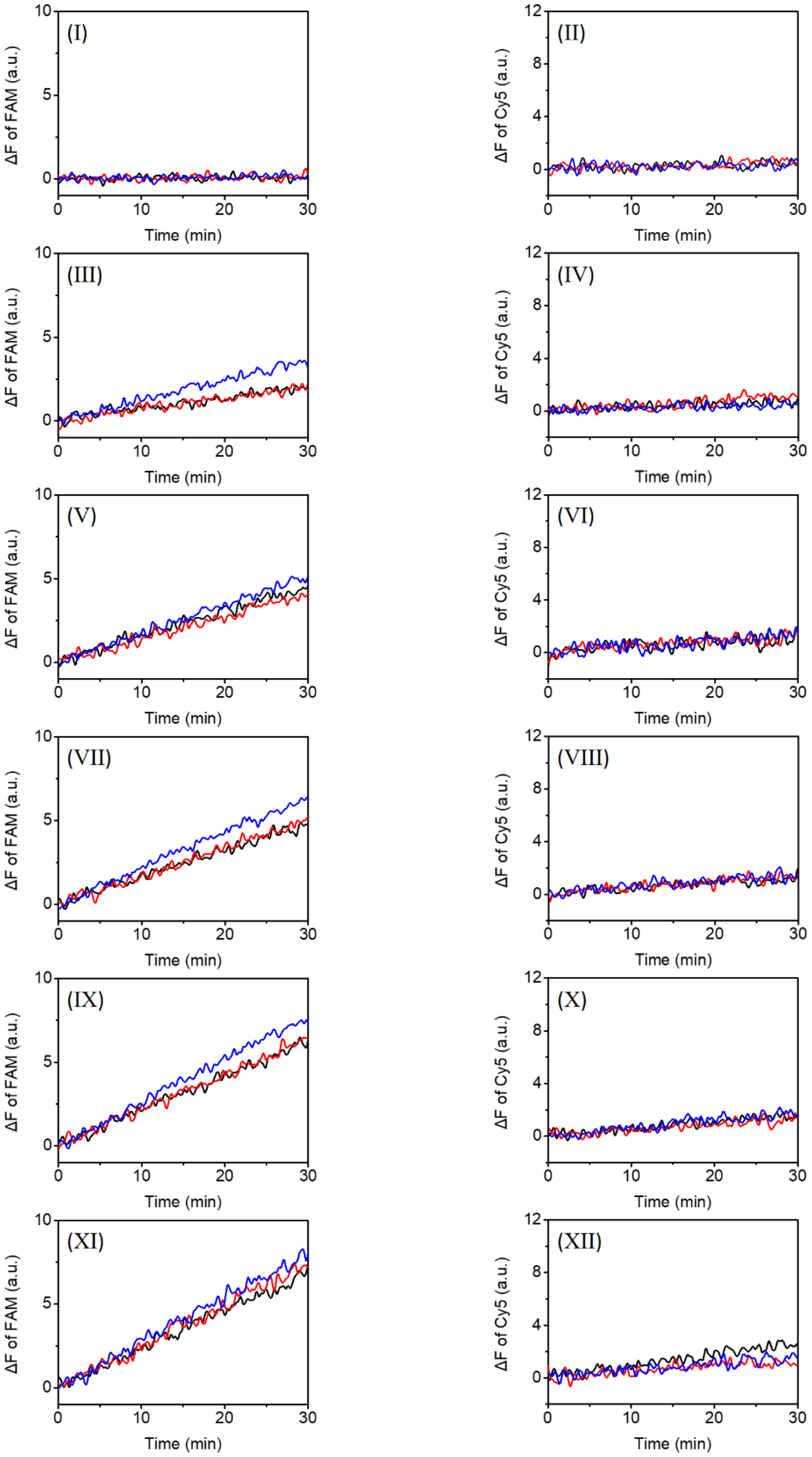

Figure S29. Time-dependent catalytic activities of DNAzyme (**1**) and DNAzyme (**2**) (DNAzyme (**2**) is inhibited) assembled by the transiently-modulated transcription machineries shown in Figure 7 in the presence of blocker (**6**) at different time-intervals: (I-II), 0 min; (III-IV), 10 min; (V-VI), 20 min; (VII-VIII), 30 min; (IX-X), 60 min; (XI-XII), 120 min. Each measurement was repeated three times (N = 3).

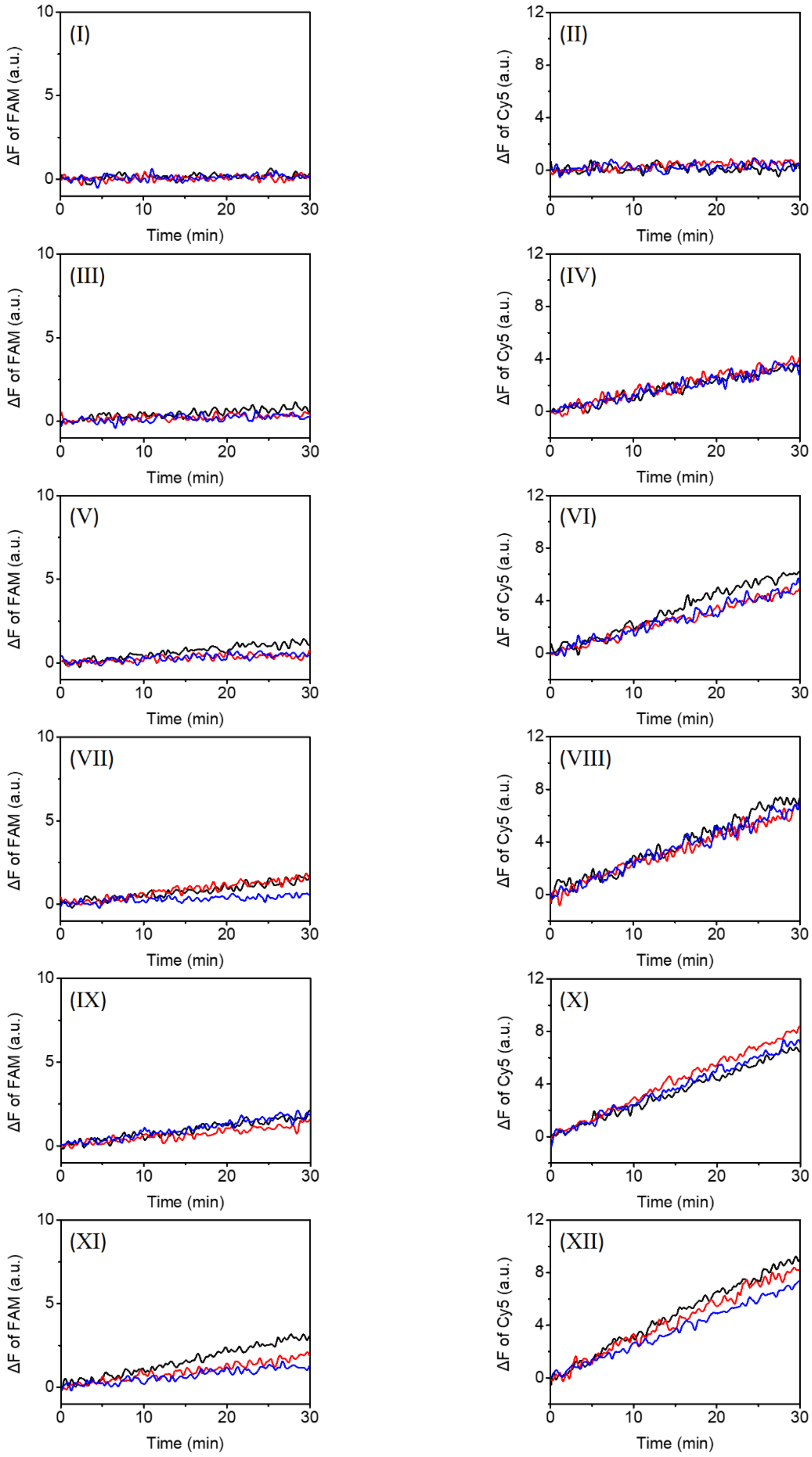

Figure S30. Time-dependent catalytic activities of DNAzyme (**1**) and DNAzyme (**2**) (DNAzyme (**1**) is inhibited) assembled by the transiently-modulated transcription machineries shown in Figure 7 in the presence of blocker (**5**) at variable time-intervals: (I-II), 0 min; (III-IV), 10 min; (V-VI), 20 min; (VII-VIII), 30 min; (IX-X), 60 min; (XI-XII), 120 min. Each measurement was repeated three times (N = 3).

Integration of two different triggered transcription machineries for the parallel dynamic transcription of two different outputs.

Integration of the Pb^{2+}-DNAzyme-modulated transcription machinery and of the strand-displacement-modulated transcription machinery for the parallel transcription of the MG-RNA output and DFHBI-RNA output.

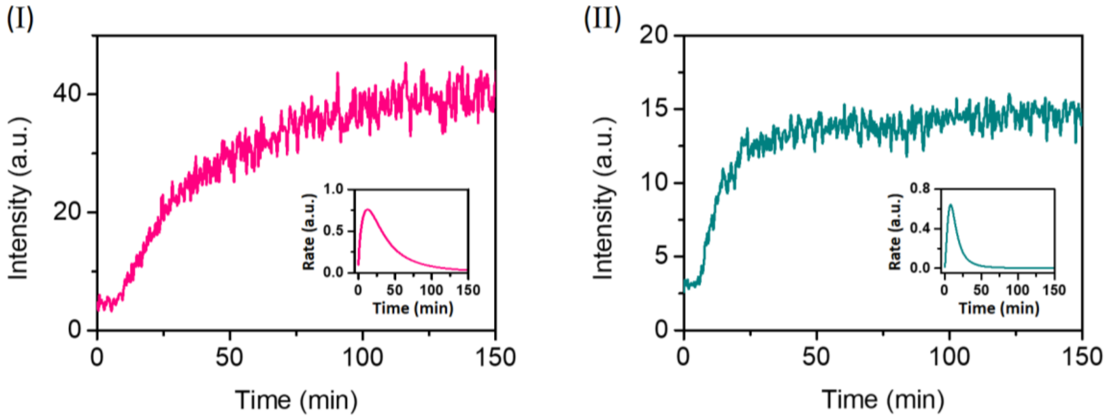

Figure S31. Panel I depicts the temporal transcription of the MG-RNA aptamer driven by the DNAzyme-modulated transcription machinery; Panel II, the temporal transcription of the DFHBI-RNA aptamer stimulated by the strand-displacement-modulated transcription machinery.

Integration of the strand-displacement-modulated transcription machinery and of the nickase-modulated transcription machinery for the parallel transcription of the MG-RNA output and DFHBI-RNA output.

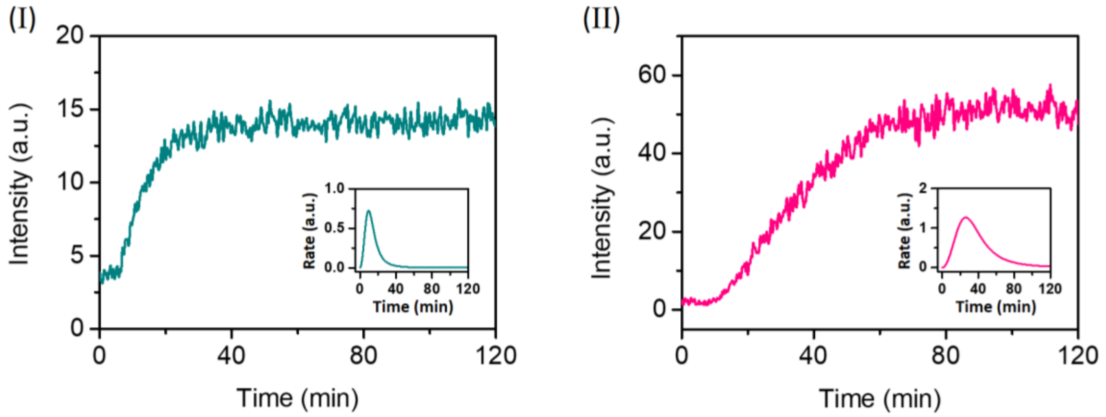

Figure S32. Panel I depicts the temporal transcription of the DFHBI-RNA aptamer driven by the strand-displacement-modulated transcription machinery; Panel II, the temporal transcription of the MG-RNA aptamer stimulated by the nickase-modulated transcription machinery.

Integration of the Pb^{2+}-DNAzyme-modulated transcription machinery and of the nickase-modulated transcription machinery for the parallel transcription of the MG-RNA output and DFHBI-RNA output.

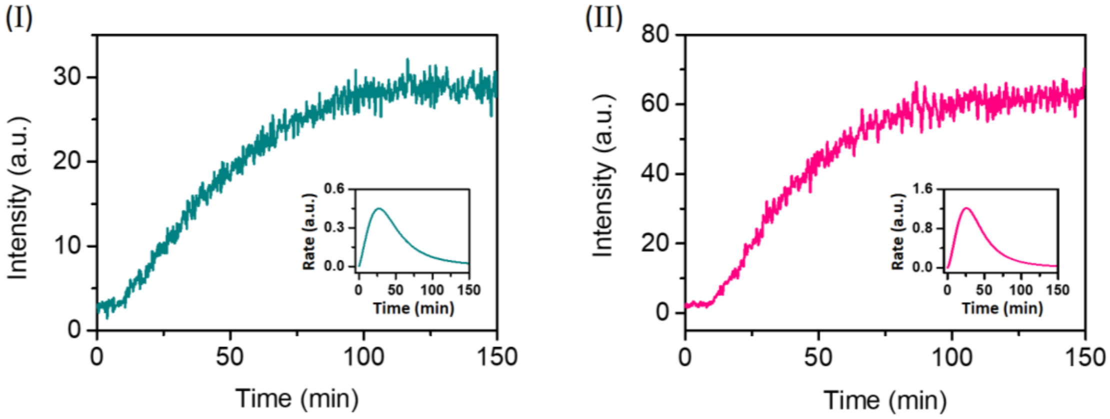

Figure S33. Panel I depicts the temporal transcription of the DFHBI-RNA aptamer driven by the DNAzyme-modulated transcription machinery; Panel II, the temporal transcription of the MG-RNA aptamer stimulated by the nickase-modulated transcription machinery.

Table S1. Rate constants derived from the computational simulation of the Pb^{2+}-DNAzyme-modulated transcription dissipative system shown in Figure 1(A).

k_1	3.04 μM^{-1} min^{-1}	k_{-2}	0.06 min^{-1}	k_5	3.00 min^{-1}
k_{-1}	0.00 min^{-1}	k_3	0.22 μM^{-1} min^{-1}	k_{-5}	2.00 μM^{-2} min^{-1}
k_2	9.09 μM^{-1} min^{-1}	k_4	0.09 min^{-1}		

Table S2. Rate constants derived from the computational simulation of the strand-displacement-modulated transcription dissipative system shown in Figure 3(A).

k_6	16.19 μM^{-1} min^{-1}	k_8	60.27 μM^{-1} min^{-1}	k_{11}	15.00 μM^{-1} min^{-1}
k_{-6}	0.00002 min^{-1}	k_{-8}	9.99 min^{-1}	k_{-11}	0.01 μM^{-1} min^{-1}
k_7	30.00 μM^{-1} min^{-1}	k_9	0.21 μM^{-1} min^{-1}	k_{12}	79.99 μM^{-1} min^{-1}
k_{-7}	0.63 min^{-1}	k_{10}	0.10 μM^{-1} min^{-1}	k_{-12}	0.10 μM^{-1} min^{-1}

Table S3. Rate constants derived from the computational simulation of the nickase-driven transcription dissipative system shown in Figure 5(A).

k_{13}	35.02 μM^{-1} min^{-1}	k_{15}	0.50 μM^{-1} min^{-1}	k_{18}	5.00 min^{-1}
k_{-13}	1.79 min^{-1}	k_{16}	21.73 μM^{-1} min^{-1}	k_{-18}	4.00 μM^{-2} min^{-1}
k_{14}	43.15 μM^{-1} min^{-1}	k_{-16}	1.67 min^{-1}		
k_{-14}	4.26 min^{-1}	k_{17}	94.78 min^{-1}		
